# Evaluation of Dietary Supplements Containing Viable Bacteria by Cultivation/MALDI-TOF Mass Spectrometry and PCR Identification

**DOI:** 10.3389/fmicb.2021.700138

**Published:** 2021-07-19

**Authors:** Petra Mohar Lorbeg, Majda Golob, Mateja Kramer, Primož Treven, Bojana Bogovič Matijašić

**Affiliations:** ^1^Department of Animal Science, Biotechnical Faculty, Institute of Dairy Science and Probiotics, University of Ljubljana, Ljubljana, Slovenia; ^2^Veterinary Faculty, Institute of Microbiology and Parasitology, University of Ljubljana, Ljubljana, Slovenia; ^3^Global Drug Development, Technical Research & Development, Biologics and Cell & Gene Therapy, Novartis, Global Project Management Office, Lek Pharmaceuticals d.d., Mengeš, Slovenia

**Keywords:** lactobacilli, bifidobacteria, dietary supplements, MALDI-TOF MS, identification, probiotic, PCR, viability

## Abstract

The insufficient quality of products containing beneficial live bacteria in terms of content and viability of labelled microorganisms is an often-reported problem. The aim of this work was to evaluate the quality of dietary supplements containing viable bacteria available in Slovenian pharmacies using plate counting, matrix-assisted laser desorption ionisation time-of-flight mass spectrometry (MALDI-TOF MS) and species- or subspecies-specific PCR with DNA isolated from consortia of viable bacteria, from individual isolates, or directly from the products. Twelve percent of the products (3 of 26) contained insufficient numbers of viable bacteria. Eighty-three of the labelled species (111 in total) were confirmed by PCR with DNA from the product; 74% of these were confirmed by PCR with DNA from viable consortium, and 65% of these were confirmed by MALDI-TOF MS analysis of colonies. Certain species in multi-strain products were confirmed by PCR with DNA from viable consortia but not by MALDI-TOF MS, suggesting that the number of isolates examined (three per labelled strain) was too low. With the exception of *Lacticaseibacillus casei* and closely related species (*Lacticaseibacillus rhamnosus* and *Lacticaseibacillus zeae*), PCR and MALDI-TOF identification results agreed for 99% of the isolates examined, although several MALDI-TOF results had lower score values (1.700–1.999), indicating that the species identification was not reliable. The species *L. zeae*, which appeared in 20 matches of the Biotyper analysis, was identified as *L. rhamnosus* by PCR. The MALDI-TOF MS analysis was also unsuccessful in detecting *Lactobacillus acidophilus* La-5 and *Bacillus coagulans* due to missing peaks and unreliable identification, respectively. Mislabelling was detected by both methods for two putative *L. casei* strains that turned out to belong to the species *Lacticaseibacillus paracasei*. PCR remains more successful in subspecies-level identification as long as the database of MALDI-TOF MS spectra is not expanded by building in-house databases. The lack of positive PCR results with viable consortia or colonies, but positive PCR results with DNA isolated directly from the products observed in 10% (11/112) of the labelled strains, suggests the presence of non-culturable bacteria in the products. MALDI-TOF MS is a faster and simpler alternative to PCR identification, provided that a sufficient number of colonies are examined. Generation of in-house library may further improve the identification accuracy at the species and sub-species level.

## Introduction

Researchers from several countries have pointed out for many years the insufficient quality of products containing beneficial live bacteria in terms of the content and viability of the labelled microorganisms (Temmerman et al., 2003; Elliott and Teversham, 2004; Huys et al., 2006; Lin et al., 2006; Matijašić and Rogelj, 2006; Morovic et al., 2016). Until the European Union Regulation on nutrition and health claims [Regulation (EC) No. 1924/2006] came into force in Europe, these bacteria were called probiotic bacteria. However, the term probiotic has since been replaced in the EU by “live bacteria,” “beneficial bacteria,” or similar terms due to the lack of approved health claims, while it continues to be used in most other regions of the world. However, these restrictions only apply to foods, including dietary supplements, and not to medicinal products, medical devices, and other products with intentionally added microorganisms. In order to distinguish between food probiotic products, including dietary supplements, and those for therapeutic use, the term “live biotherapeutic products” was introduced by the FDA (2012) and since 2019 has also been recognised by the European Directorate for the Quality of Medicines and Healthcare (Cordaillat-Simmons et al., 2020).

In Slovenia, the quality of dietary supplements containing live bacteria from Slovenian pharmacies, marketed as “probiotic” until 2018, has been monitored for almost two decades (Matijašić and Rogelj, 2006; Bogovič Matijašić et al., 2010; Lorbeg and Matijašić, 2017). Our study published in 2017 showed that the overall quality of products sold in Slovenian pharmacies improved slightly, as 42% of them complied with the label, compared to only 25% in the previous study in 2009 (Lorbeg and Matijašić, 2017). The need for stricter control of orally administered probiotic formulations, which should contain the specified number of viable bacteria and be free of contaminating microorganisms, was also recently highlighted by the European Society for Paediatric Gastroenterology Hepatology and Nutrition Working Group for Probiotics and Prebiotics (Kolacek et al., 2017). Although significant improvements in the quality of these products have been observed in other markets in recent years, deficiencies in the content and viability of the declared microorganisms are still regularly reported (Lewis et al., 2016; Chen et al., 2017; Ansari et al., 2019; Di Pierro et al., 2019; Korona-Glowniak et al., 2019; Seol et al., 2019; Aldawsari et al., 2020; Dioso et al., 2020; Kesavelu et al., 2020; Shehata and Newmaster, 2020; Vermeulen et al., 2020).

Products containing multiple strains present a particular challenge, as not all strains need to be present in the same quantity at the time of production and can vary greatly in viability during storage. In addition to conventional cultivation-based and PCR-based methods for viability assessment and identification, more advanced methods are increasingly being used, such as next-generation sequencing, terminal restriction fragment length polymorphism, and matrix-assisted laser desorption ionisation time-of-flight mass spectrometry (MALDI-TOF MS) (Angelakis et al., 2011; Lewis et al., 2016; Morovic et al., 2016; Patro et al., 2016; Čanžek Majhenič et al., 2018; Huang and Huang, 2018; Vecchione et al., 2018; Celandroni et al., 2019; Seol et al., 2019; Dioso et al., 2020; Vermeulen et al., 2020). Although some of them, such as viability PCR methods and advanced flow cytometry methods, are rapid and provide high-quality results, they may not be as useful for routine quality control because they require expensive equipment and chemicals as well as well-trained personnel.

Bacterial identification using MALDI-TOF is based on the comparison of protein mass spectra obtained from whole bacterial cells (Moore et al., 2011; Singhal et al., 2015). This method has already been recognised as an attractive tool for clinical isolate identification, microbial diversity studies, and other applications. With the improved databases, it is now increasingly used also for the analysis of milk and dairy products (Angelakis et al., 2011; Duskova et al., 2012; Nacef et al., 2017; Gantzias et al., 2020) but has not been widely used for the quality control of dietary supplements with added live beneficial bacteria (probiotic products) (Singhal et al., 2015; Čanžek Majhenič et al., 2018). A few reports on the use of MALDI-TOF MS for the analysis of dietary supplements include the identification of bifidobacteria, lactic acid bacteria (LAB), *Bacillus* species, and *Saccharomyces boulardii* in various products available on the EU market and in the United States (Bunesova et al., 2014; Vecchione et al., 2018; Ansari et al., 2019; Celandroni et al., 2019).

Previous studies in Slovenia and elsewhere have shown that there are inconsistencies between reported and observed data on the number and type of live bacteria in products. In this study, dietary supplements containing live bacteria available in Slovenian pharmacies were evaluated for compliance with the declared content of microbial species and amounts within the declared shelf-life of the product using conventional cultivation methods and identification of colonies by analysis of the mass spectra of cell proteins with MALDI-TOF MS. In addition, the main objective was to evaluate the applicability of MALDI-TOF MS in the identification of labelled bacteria in the products studied compared to the PCR approach.

## Materials and Methods

### Products

Dietary supplements for the general population (*n* = 17; marked as 1–17) or children (*n* = 9; marked as 18–26) were purchased in Slovenian pharmacies in March 2019. They were stored under the conditions indicated on the labels until analysis.

### Determination of Viable Counts (CFU/g)

The products in the powdered or oil suspension formulations were aseptically diluted and homogenised in the buffered peptone water (10% w/w) (Merck, Darmstadt, Germany). The products in tablet form were crushed to a fine powder before homogenization. The homogenised samples were allowed to stand at room temperature for 30 min to rehydrate. The appropriate dilution was spread on agar media plates as follows: TOS (Yakult, Tokyo, Japan) with mupirocin (50 mg/L) (AppliChem GmbH, Germany) for bifidobacteria, MRS (pH 6.2) for lactobacilli and *Bacillus*, ROGOSA (pH 5.5) with glacial acetic acid (0.13%) for lactobacilli in mixed formulations, M17 for lactococci and *Streptococcus* (*S.*) *salivarius* subsp. *thermophilus*, CATC for enterococci, and YGC for yeasts. Microbiological media were purchased from Merck (Darmstadt, Germany) except where otherwise indicated. Incubation followed for bifidobacteria and lactobacilli at 37°C for 72 h, anaerobically (GENbox anaer, Bio-Mérieux, Marcy l’Etoile, France); *S. salivarius* subsp. *thermophilus*, aerobically at 37°C for 24 h; lactococci, aerobically at 30°C for 72 h; *Bacillus*, aerobically at 37°C for 5 days; and yeasts, aerobically at 25°C for 72 h.

### MALDI-TOF MS Identification

In general, the number of colonies examined by MALDI-TOF was three times the number of labelled species. Putative representatives of different species (three colonies per labelled strain) were isolated from different agar media selected based on label information. Colonies were selected based on the morphological characteristics of the colonies when observed, otherwise randomly from a portion of the agar plate.

Colonies were transferred onto a steel plate (MSP 96 target polished steel BC, Bruker, Germany) and air-dried (direct spotting method). One microlitre of HCCA matrix solution (α-cyano-4-hydroxycinnamic acid; Bruker Daltonics, Bremen, Germany) was applied to each sample and further processed according to the manufacturer’s instructions (Microflex LT system TM 1.1, Bruker Daltonics, Germany). Identification was performed using MALDI Biotyper 3.1 software and library (version 3.1.66; Bruker Daltonics). Calibration of the mass spectrometer was performed with the Bruker’s bacterial test standard (*Escherichia coli* DH5α extracts with the additional proteins RNase A and myoglobin, Bruker Daltonics). The results of the comparative analysis of the obtained spectra with the reference spectra were expressed as confidence score values and associated colour codes. The score values ≥ 2.300 were considered as highly probable species identification. Score values ranging from 2.000 to 2.299 and from 1.700 to 1.999 meant confident genus identification/probable species identification and probable genus identification, respectively. In case of unreliable/unsatisfactory results (score values below 1.7), the extended direct transfer method with 70% formic acid was used.

### Bacterial DNA Isolation

DNA was isolated directly from the products and from a mixture of colonies grown on selective media (i.e., viable consortia). To isolate DNA directly from the product, 50 mg of a sample was transferred to a microtube and resuspended in 0.4 ml of TE buffer containing 4 mg of lysozyme (Sigma Chemical, St. Louis, MO, United States) and 4 μl of mutanolysin (2,500 U/ml) (Sigma Chemical, St. Louis, MO, United States). After 1 h of incubation at 37°C, DNA was isolated using Maxwell 16 Tissue DNA Purification Kit (Promega, Madison, WI, United States) according to the manufacturer’s protocols. In addition, DNA was also isolated from the mixture of colonies washed from the selective media by pipetting 2 ml of physiological saline onto the surface of the media, then scraping the colonies from the surface with a disposable L-shaped cell spreader, and transferring 1 ml of the suspension to a microcentrifuge. The suspension was centrifuged at 12,000 rpm for 2 min, and DNA was isolated from the pellet as described above.

The colonies selected for MALDI-TOF MS analysis were first streaked onto appropriate selective media and incubated under the conditions described above. Grown colonies were scraped from the media, resuspended in 0.4 ml TE buffer, and stored in the freezer. Prior to isolation, 0.1 ml TE buffer containing 5 mg lysozyme and 5 μl mutanolysin (2,500 U/ml) was added and incubated for 1 h at 37°C. The suspension was then centrifuged at 12,000 rpm for 2 min, and the supernatant was removed. DNA was further isolated using the Isolate II Genomic DNA Kit (Bioline Reagents Ltd., London, United Kingdom) according to the manufacturer’s instructions.

### Species-Specific PCR

PCR specific for particular species or subspecies was performed with DNA isolated directly from the products or from viable consortia, i.e., colonies scraped from agar plates. The reaction mixtures (20 μl) contained GoTax flexi buffer (Promega), 1.5–7 mM MgCl_2_, 0.5 μM oligonucleotide primers, 0.1 mM dNTP, 0.025 U/μl Taq polymerase, and 2 μl DNA. PCR reactions were performed on Simplism^TM^ Thermal Cycler (Thermo Fisher Scientific, United States) using specific primers as described previously: *Lacticaseibacillus casei*, *Lacticaseibacillus paracasei* (Ward and Timmins, 1999), *L. casei*, *L. paracasei*, *Lacticaseibacillus rhamnosus* (Bottari et al., 2017), *Lactobacillus plantarum*, *Lactobacillus salivarius* (Chagnaud et al., 2001), *L. rhamnosus*, *Lactobacillus fermentum*, *Lactobacillus reuteri*, *Lactobacillus acidophilus* (Walter et al., 2000), *Lactobacillus delbrueckii* subsp. *bulgaricus* (Torriani et al., 1999), *Lactobacillus gasseri* (Song et al., 2000), *Lactococcus lactis* (Barakat et al., 2000), *Bifidobacterium bifidum*, *Bifidobacterium infantis* (Matsuki et al., 1999), *Bifidobacterium longum*, *Bifidobacterium animalis* subsp. *lactis* (Malinen et al., 2003), *B. animalis* (Junick and Blaut, 2012), *Bifidobacterium breve* (Kwon et al., 2005), *Saccharomyces cerevisiae* (Josepa et al., 2000), *Bacillus coagulans* (Liu et al., 2010), *Enterococcus faecium* (Dutkamalen et al., 1995), and *S. salivarius* subsp. *thermophilus* (Tilsala Timisjarvi and Alatossava, 1997). The primers and reaction conditions are listed in Supplementary Table 1. The presence and size of PCR amplicons were checked by agarose gel electrophoresis on 1.2% agarose gels (agarose Sigma) using TAE buffer (Sigma) and Gene Ruler, 1 kb DNA Ladder (Thermo Scientific). Gels were stained with SYBR Safe (Invitrogen, OR, United States), visualised under UV, and documented using the UVITEC system (Cambridge, United Kingdom).

### 16S rDNA Sequencing

The DNA of selected strains, extracted as described above, was amplified by PCR using primers 27F (5′-AGAGTTTGATCCTGGCTCAG-3′) and 1495R (5′-CTACGGCTACCTTGTTACGA-3′) (Yu et al., 2011). The reaction mixture was the same as described above for species-specific PCR. The amplification protocol included 2 min of denaturation at 95°C, 30 cycles of denaturation (95°C, 1 min), annealing (59°C, 1 min), elongation (72°C, 2 min), and final elongation for 5 min at 72°C. Sanger sequencing was performed by Microsynth (Balgach, Switzerland). The sequences were analysed using National Center for Biotechnology Information database and BLAST algorithm.^1^

## Results

The products examined comprised those for the general population (*n* = 17) and those for children (*n* = 9). Ten of them (38.5%) contained only one strain, two (7.7%) contained two strains, two (7.7%) contained three strains, and nine products (34.6%) contained more than three strains (4–14) belonging to different genera, i.e., *Lactobacillus*, *Bifidobacterium*, *Lactococcus*, and newly introduced genera derived from previous *Lactobacillus*, i.e., *Lacticaseibacillus*, *Limosilactobacillus*, *Lactiplantibacillus*, and *Ligilactobacillus* (Zheng et al., 2020). It should be noted that the strains indicated on the labels (Supplementary Table 2) still bear the old species names, as the products were obtained before the publication of the new classification of the former genus *Lactobacillus* in 2020 (Zheng et al., 2020).

### Determination of Viable Counts (CFU/g)

**FIGURE 1:**
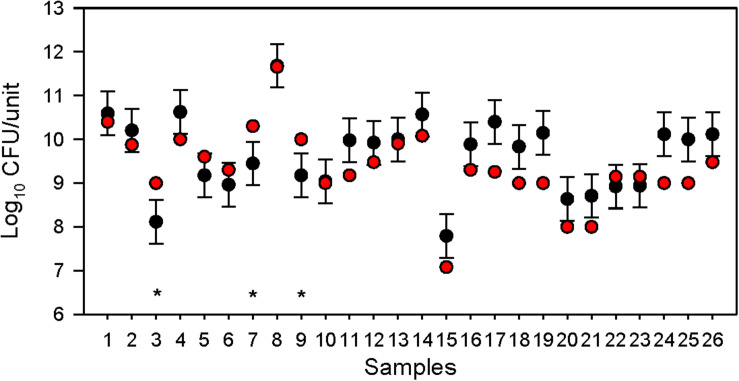
Compliance of 26 dietary supplements with the labelled number of colony-forming units (CFU; bacteria). Black dots, number (CFU/unit of product) determined by plate counting; whiskers, 0.5 log_10_ acceptable accuracy; red dots, labelled number of CFU/unit of product; *, non-compliance with the labelled number of CFU.

Compliance with the labelled number of viable bacteria was not related to the date of purchase, as eight products that were not fully adequate in terms of the number of viable bacteria were not close to the end of shelf-life, but at least 12 months remained until the end of shelf-life. For five products, the number of colony-forming units per gram (CFU/g) differed by less than 0.5 log units, while for three products the viable count was 0.5–1 log unit lower than indicated (Figure 1 and Supplementary Table 2).

The results which were <0.5 log below the indicated CFU/dose were not considered a serious deviation from the indicated concentration since we should also take into account the usual deviations of the plate counting method and since the media and protocols of sample preparation may not have been optimal for certain strains. In plate counting, indeed up to about ±0.5 log_10_ is often considered as acceptable accuracy (ISO, 2003, 2010; Jackson et al., 2019). Overall, three products out of the 26 tested (12%) can be considered inadequate in terms of viability.

### Assessment of the Presence of Labelled Species by Species-Specific PCR

To assess the presence of the species indicated on the labels, three approaches were used. First, individual colonies were collected from agar plates and analysed by MALDI-TOF MS and by species-specific PCR using primers specific to the species listed on the labels. Second, DNA was isolated from a mixture of colonies grown on specific agar media (DNA from viable consortia) and subjected to species-specific PCR. Third, total (metagenomic) DNA was isolated from the products and analysed by species-specific DNA.

Overall, 83% of the labelled species (111 total) were confirmed by PCR with DNA from the product, 74% of them were confirmed by PCR with DNA from viable consortium, and 65% of them were confirmed by MALDI-TOF MS analysis of colonies (Table 1). When specific species were detected in the products by PCR on isolates, the presence of these species was generally also detected in total DNA from the product and in DNA from viable consortia (Table 1). Two exceptions were *B. breve* in product 8 and *L. casei* species in product 3, which could not be detected by PCR in DNA from viable consortia or from the products, and *L. acidophilus* in product 17 which could not be detected in DNA from the product, Conversely, in some cases, such as *B. longum*, *L. paracasei*, *L. salivarius* in product 1, *B. bifidum* in product 2, etc., the species was confirmed only by PCR with total DNA, which may indicate that the representatives of these species are present in the product in a non-culturable state or in low numbers. DNA from product 3 and DNA from viable consortia were also amplified with *L. paracasei* primers, which should not generate amplicons with *L. casei* and *L. rhamnosus* (Ward and Timmins, 1999), indicating that mislabelling of *L. casei* in this product is possible. Similarly, mislabelling for *L. casei* is likely in products 12 and 25, where the presence of *L. paracasei* was confirmed by PCR on individual isolates and metagenomic DNA.

**TABLE 1:** Results of the assessment of the presence of labelled species or subspecies in 17 dietary supplements for adults (A1–A17) and of 9 dietary supplements for children (C1–C9) by PCR using DNA isolated from products and from viable consortia (colonies) and by MALDI-TOF MS analysis of individual colonies (three per labelled strain).

Product	Labelled species (as stated on the labels)	Species-specific PCR (DNA from product)	Species-specific PCR (DNA from viable consortia)	Species-specific PCR (DNA from single colonies)	MALDI-TOF MS (single colonies)
1	*L. acidophilus*	+	+	+	+
	*L. plantarum*	+	+	+	+
	*L. rhamnosus*	+	+	−	−
	*B. breve*	−	−	−	−
	*L. salivarius*	+	−	−	−
	*B lactis*	+	+	+	+
	*L. casei*	+	+	+	+ (also *zeae*+)
	*L. paracasei*	+	−	−	−
	*S. thermophilus*	+	+	+	+
	*B. longum*	+	−	−	−
2	*L. casei*	− (*para*+)	− (*para*+)	− (*para*+)	− (*para*+)
	*L. acidophilus*	+	+	+	+
	*L. paracasei*	+	+	+	+
	*B. lactis*	+	+	+	+
	*L. salivarius*	+	+	+	+
	*Lc. lactis*	+	+	+	+
	*B. lactis*	+	+	+	+
	*L. plantarum*	+	+	+	+
	*B. bifidum*	+	−	−	−
3	*L. acidophilus*	+	+	+	+
	*L. casei*	− (*para*+)	− (*para*+)	−	+
	*L. plantarum*	+	−	−	−
	*L. reuteri*	+	+	−	−
	*L. rhamnosus*	+	+	+	+
	*B. longum*	+	+	+	+
	*S. thermophilus*	+	+	−	−
4	*B. animalis* subsp. *lactis*	+	+	+	+
	*L. paracasei*	+	+	+	+
	*B. breve*	+	+	+	±
	*L. gasseri*	+	+	+	+
	*L. rhamnosus*	+	+	+	±
	*L. acidophilus*	+	+	+	+
	*L. plantarum*	+	+	−	−
	*B. longum* subsp. *longum*	+ (*longum*)	−	−	−
	*B. bifidum*	+	+	+	+
	*L. casei*	+	+	+	+
	*L. reuteri*	+	+	−	−
	*Lc. lactis*	+	+	+	+
	*B. longum* subsp. *infantis*	+	−	−	−
5	*L. acidophilus*	+	+	+	+
	*B. lactis*	+	+	+	+
	*L. plantarum*	+	−	−	−
	*B. breve*	−	−	−	−
6	*Bac. coagulans*	+	+	+	−
7	*L. acidophilus* DDS-1	+	+	+	+
	*B. lactis*	+	+	+	+
	*L. plantarum*	+	−	−	−
	*L. rhamnosus*	+	+	+	+
	*L. casei*	+	−	−	− (*zeae*±)
	*B. longum*	+	−	−	−
	*S. thermophilus*	+	+	+	+
8	*S. thermophilus*	+	+	+	±
	*B. breve*	−	−	+	±
	*B. longum*	+	−	−	−
	*B. infantis*	−	−	−	−
	*L. acidophilus*	+	+	+	+
	*L. plantarum*	+	+	+	+
	*L. paracasei*	+	+	+	+
	*L. delbrueckii* subsp. *bulgaricus*	−	−	−	−
9	*L. rhamnosus*	+	+	+	±
10	*L. reuteri*	+	+	+	+
11	*Sacch. boulardii*	− (*cer*+)	− (*cer*+)	− (*cer*+)	− (*cer*+)
	*L. acidophilus*	+	+	+	+
	*B. breve*	+	+	+	+
	*B. infantis*	+	+	−	−
	*B. longum*	+	+	−	−
12	*B. animalis*	+	+	+	+
	*L. acidophilus*	+	+	+	+
	*L. salivarius*	+	+	+	+
	*E. faecium*	+	+	+	+
	*Lc. lactis*	+	+	+	+
	*L. casei*	− (*para*+)	− (*para*+)	− (*para*+)	− (*para*+)
13	*L. acidophilus*	+	+	+	+
	*L. paracasei*	+	+	+	±
	*L. rhamnosus*	+	+	−	−
	*E. faecium*	+	+	−	−
	*L. salivarius*	+	+	+	+
	*L. plantarum*	+	+	+	+
	*B. bifidum*	−	−	−	−
	*B. lactis*	+	+	+	+
	*B. longum*	−	−	−	−
14	*L. rhamnosus*	+	+	+	±
15	*L. gasseri*	+	+	+	+
	*B. infantis*	+	+	+	+
	*E. faecium*	+	+	+	+
16	*L. acidophilus*	+	+	+	−
	*B. animalis* subsp. *lactis*	+	+	+	+
17	*L. plantarum*	−	+	+	+
	*L. fermentum*	−	+	−	−
	*L. acidophilus*	−	+	+	+
	*L. reuteri*	−	+	−	−
	*L. rhamnosus*	−	+	−	−
	*B. bifidum*	−	−	−	−
18	*Bif. animalis* subsp. *lactis*	+	n.d.	n.d.	+
19	*Bif. animalis* subsp. *lactis*	+	n.d.	n.d.	+
20	*L. reuteri*	+	n.d.	n.d.	+
21	*L. reuteri*	+	n.d.	n.d.	+
22	*L. rhamnosus*	+	n.d.	n.d.	+
23	*L. rhamnosus*	+	n.d.	n.d.	+
24	*L. rhamnosus*	+	n.d.	n.d.	+
	*Bif. animalis* subsp. *lactis*	+	n.d.	n.d.	+
25	*L. casei*	− (*para*+)	n.d.	n.d.	± (*para*+)(*zeae*±)
	*L. rhamnosus*	+	n.d.	n.d.	+
	*Bif. breve*	+	n.d.	n.d.	+
	*Bif. longum* subsp. *infantis*	+	n.d.	n.d.	+ (*B. longum*)
	*L. delbrueckii* subsp. *bulgaricus*	+	n.d.	n.d.	−
	*S. thermophilus*	+	n.d.	n.d.	+
	*L. acidophilus*	−	n.d.	n.d.	− (*gall*±)(*helv*±)
26	*Lc. Lactis*	+	n.d.	n.d.	+
	*Bif. animalis* subsp. *lactis*	+	n.d.	n.d	+ (*B. animalis*)
	*Bif. bifidum*	+	n.d.	n.d.	−

**Bac.*, *Bacillus*; *B.*, *Bifidobacterium*; *E.*, *Enterococcus*; *L.*, *Lactobacillus* [in accordance with the new taxonomic classification*;*Zheng et al. (2020); also *Lacticaseibacillus*, *Lactiplantibacillus*, *Limosilactobacillus*, *Ligilactobacillus*]; *Lc.*, *Lactococcus*; *S. thermophilus*, *Streptococcus salivarius* subsp. *thermophiles*; + (MALDI-TOF MS), species confirmed with at least one isolate; − (MALDI-TOF MS), species not confirmed; ± (MALDI-TOF MS), score value 1.700–1.999; − (*cer*+), negative result for *Sacch. boulardii*, positive result for *Sacch. cerevisiae*; − (*para*+), negative result for *Lacticaseibacillus casei*, positive result for *Lacticaseibacillus paracasei*; − (*zeae*+), negative result for *Lacticaseibacillus casei*, positive result for *Lacticaseibacillus zeae*; − (*gall*+)(*helv*+), negative result for *Lactobacillus acidophilus*, positive result for *Lactobacillus gallinarum* and *Lactobacillus helveticus*; (*longum*), PCR was specific for *B. longum* species, not for *B. longum* subsp. *longum*; n.d., not determined.*

Interestingly, in one of the products (sample 17), we could not amplify any of the target sequences by PCR with DNA isolated directly from the product, although DNA isolation was repeated three times and additional purification of DNA was performed to remove possible PCR inhibitors, while five labelled species were detected by PCR with DNA from viable consortia.

### Comparison of MALDI-TOF MS and PCR on Individual Colonies

The best matches of the analysis of MALDI-TOF MS results by Biotyper together with the score values indicating the probability of correct identification are shown in Supplementary Table 3 and the overview in Tables 1,2 and Supplementary Table 4. Sixty-six percent (238/362) of the colonies were identified as highly probable or probable species (score value ≥ 2.000, highlighted in green) and 30% (107/362) were with probable genus identification (score value 1.700–1.999), while 17/362 (5%) of the samples could not be identified due to the absence of peaks (11 samples) or due to non-reliable identification (6 samples).

**TABLE 2:** Overview of the validation of MALDI-TOF MS (Biotyper) identification of 292 isolates (three isolates per labelled strain) from 17 dietary supplements (1–17) by PCR.

Result of identification by MALDI-TOF MS (Biotyper)	Score value ≥ 2.000	Score value 1.700–1.999	Species-specific PCR
*B. animalis*	45	12	1 n.d., all others *B. animalis* subsp. *lactis*
*B. breve*	8	6	All *B. breve*
*B. longum*	1	2	All *B. longum*
*B. bifidum*	1	/	All *B. bifidum*
*B. infantis*	3	/	All *B. infantis*
*E. faecium*	4	5	All *E. faecium*
*L. acidophilus*	54	5	1 n.d., all others *L. acidophilus*
*L. casei*	3 (one of them *L. casei* or *L. zeae*)	1	Two (one score value 2.203, the other score value 2.15 or 2.168, *L. casei* or *L. zeae*) *L. casei*; one (score value 2.042) *L. rhamnosus*; one (score value 1.847) *L. rhamnosus*
*L. gasseri*	6	/	1 n.d., all others *L. gasseri*
*L. paracasei*	26	2	All *L. paracasei*
*L. plantarum*	16	17	All *L. plantarum*
*L. reuteri*	6	/	5 *L. reuteri*, 1 n.d.
*L. rhamnosus*	27	8	All *L. rhamnosus*
*L. salivarius*	5	/	All *L. salivarius*
*L. zeae* (*L. rhamnosus* and *L. casei* on the labels)		12	12 *L. rhamnosus*
*Lc. Lactis*	9	/	All *Lc. Lactis*
*Sacch. cerevisiae*	1	5	All *Sacch. Cerevisiae*
*S. thermophilus*	3	5	All *S. thermophilus*
Not reliable identification (score values < 1.700)	6		3 *Bac. coagulans*
			1 n.d.
			1 *L. plantarum*
			1 *L. rhamnosus*
No peaks detected	11		6 *L. acidophilus*
			3 *B. lactis*
			2 *E. faecium*

*n.d., not determined.*

Overall, highly probable species identification (score value ≥ 2.300) was not frequently observed. Only 33 of 362 (9%) had a score value above 2.3, and the best match was not restricted to specific species. In general, not only the “green” results of MALDI-TOF MS but also most results with score values of 1.700–1.999 (highlighted in yellow) were confirmed by species-level PCR (Supplementary Table 3).

The matching of identification results for individual colonies with MALDI-TOF MS and PCR was generally good but with a few exceptions (Tables 1, 2 and Supplementary Table 3). Two putative *L. casei* in product 3 (score values 2.042 and 1.847, respectively) were not confirmed as *L. casei* by PCR but were identified as *L. rhamnosus*. This species was also indicated on the label of product 3. Twelve isolates were identified as *Lacticaseibacillus zeae* by MALDI-TOF MS, but with score values of 1.700–1.999, indicating low reliability of results at the species level. Considering that *L. rhamnosus* species was not found among the best matches, it is likely that these isolates actually belong to *L. zeae* species but were mislabelled – which is not unusual considering that these two species are closely related and that the current type strain ATCC 15820/DSM 20178 of *L. zeae* (Liu and Gu, 2020) has been reclassified a few times. However, PCR with primers PrI and RhaII, which are specific for *L. rhamnosus* and do not amplify the DNA of *L. zeae* (Walter et al., 2000), yielded positive results for these 12 isolates. Furthermore, the *L. rhamnosus* species identity was confirmed also by PCR using the primers and conditions of Bottari et al. (2017) as well as by 16S rDNA sequencing (Supplementary Table 3).

Eleven isolates showed no peaks but could be identified by PCR. Among the isolates that could not be reliably identified by MALDI-TOF MS, while PCR analysis confirmed the labelled species, were strain *L. acidophilus* La-5 from product A16 and *B. coagulans* from product 8. In the case of strain La-5, no peaks were observed, while in the case of *B. coagulans*, the score value was less than 1.700.

The results of MALDI-TOF MS identification are not considered reliable at the subspecies level, so the best matches obtained by Biotyper with the presumed *B. animalis* ssp. *lactis* were shown as *B. animalis*. In fact, the spectra of the isolates of this species typically matched *B. animalis* subsp. *lactis* DSM 10140 (type strain) in the first place. All these isolates were confirmed by PCR to belong to the subspecies *B. animalis* subsp. *lactis* (Tables 1, 2 and Supplementary Table 3). In the case of *S. salivarius* subsp. *thermophilus*, the best matches in all cases were presented as *S. salivarius* subsp. *thermophilus*. *B. longum* representatives belonging to the subspecies *B. longum* subsp. *longum* or *B. longum* subsp. *infantis* were labelled either at the subspecies level (correct only in product 4; in another five products as *B. infantis*) or at the species level (as *B. longum*) in nine products. In one of the products (sample 15) with *B. infantis* on the label, this subspecies was confirmed by MALDI-TOF MS as *B. longum* subsp. *infantis*, while in another product (sample 25), the result was shown as *B. longum* (Supplementary Table 3). Results for the presence of *B. longum* subsp. *longum* were reported exclusively as *B. longum* in those cases where they were positive, but in fact in all these cases one of the *B. longum* subsp. *longum* reference strains from the Biotyper database was reported as the best result, so we can assume that identification was indeed successful at the subspecies level.

The identity of *B. longum* subsp. *infantis* was also confirmed by a PCR assay that was able to distinguish between the subspecies *B. longum* subsp. *longum* and *B. longum* subsp. *infantis* (Matsuki et al., 1999), while the *B. longum*, where labelled (including a *B. longum* subsp. *longum* strain), was analysed by PCR using only the primers that generated positive results in both subspecies (Malinen et al., 2003).

Fifty-six colonies were identified with higher or lower probability as *L. acidophilus*, with score values ranging from 1.903 to 2.494. In accordance with the Biotyper recommendations, it should be taken into account that the species *L. acidophilus*, *Lactobacillus amylovorus*, *Lactobacillus gallinarum*, and *Lactobacillus kitasatonis* of the genus *Lactobacillus* have very similar patterns, making it difficult to distinguish between these species. Among the best matches, *L. gallinarum* was found twice and *L. acidophilus* 70 times. Nevertheless, PCR confirmed the species *L. acidophilus* for all colonies tested and confirmed the presence of this species in 13 products in which it was labelled.

## Discussion

Viability assessment by plate counting showed certain improvements in the quality of these products available in Slovenian pharmacies compared to previous studies. While in the 2017 study, which included 17 products, 29% of the products were found to have an insufficient number of CFU/dose and another 24% of the products had a slightly lower number (<0.5 CFU/dose) (Lorbeg and Matijašić, 2017), the present study showed an insufficient number in 12% (3/26) of the products and a partially insufficient number (<0.5 CFU/dose) in 19% of products. The improvements are even more significant compared to the situation in 2010 when 65% (13 out of 20 tested) of dietary supplements or medicinal products did not contain sufficient CFU of the labelled bacteria (Matijašić and Rogelj, 2006; Bogovič Matijašić et al., 2010).

Despite some limitations and drawbacks of plate counting, such as lack of differentiation among species, counting a chain as 1 CFU, time-consuming and labour-intensive procedures, lack of standardised culture media, etc., this approach is still predominantly used in the control of products containing intentionally added viable microorganisms (Vinderola et al., 2019). In addition, advanced formulations or strain characteristics often require optimised protocols, and protocols developed by manufacturers are often not available. Nevertheless, several recent studies conducted in different parts of the world, such as China, United States–Canada, Philippines–Korea, Italy–France, and Poland, have demonstrated through plate counting that products that do not comply with labels are still regularly found in the market (Chen et al., 2017; Di Pierro et al., 2019; Korona-Glowniak et al., 2019; Dioso et al., 2020; Shehata and Newmaster, 2020). Chen et al. (2017) reported that viable cells could not be recovered from two (25%) probiotic supplements from the Chinese market. In four of seven dietary supplements (57%) from the Italian and French markets that claimed a beneficial effect on the urogenital tract, the number of total viable count did not match the label (57%) (Di Pierro et al., 2019). Five out of 10 (50%) tested products available in Poland did not reach the number of viable bacteria claimed by the manufacturer (Korona-Glowniak et al., 2019). One in 10 (10%) probiotic preparations available for consumption by children in the Republic of the Philippines and the Republic of Korea contained a lower number of viable microorganisms than claimed on the label (Dioso et al., 2020). The number of viable microorganisms determined in 72 samples of probiotic products from the United States and Canada was lower than labelled in 5 samples (6.9%), including one product that had no viable cells (Shehata and Newmaster, 2020).

Our observation that the adherence to the number of viable bacteria was not associated with the date of purchase is consistent with the study by Morovic et al. (2016) who found no significant correlation between the estimated number of viable bacteria relative to the declared number of viable bacteria and the time to expiration (*R*-square was 0.02547, and *P*-value was 0.1806) (Morovic et al., 2016).

In our previous studies (Bogovič Matijašić et al., 2010; Lorbeg and Matijašić, 2017), we also pointed out the absence of certain species among the viable microbiota from the products determined by PCR performed on DNA retrieved from individual colonies and from the mixture of colonies grown on dedicated agar media (DNA from viable consortia). In contrast to previous studies, this study also determined the presence of labelled species by analysing individual colonies using MALDI-TOF MS. In addition, species-specific PCR of the same colonies and of the total DNA from product or viable consortia was performed.

The incorrect naming of *B. longum* subsp. *infantis* as *B. infantis* observed in this study still appears to be very common and has been reported previously. The two subspecies *B. longum* subsp. *infantis* and *B. longum* subsp. *longum* are difficult to differentiate taxonomically, thus misidentifications and mislabelling are common (Lewis et al., 2016; Morovic et al., 2016; Patro et al., 2016; Shehata and Newmaster, 2020). In this study, *B. longum* has been examined by PCR, with the primers yielding positive results for both subspecies (Malinen et al., 2003), while *B. longum* subsp. *infantis* (products 4, 11, 15, 8) has been confirmed by PCR specific for this subspecies (Matsuki et al., 1999). However, it appears that both subspecies could also be distinguished by MALDI-TOF MS, considering the best matches. The MALDI-TOF MS results showed that all strains labelled as *B. longum* indeed had high similarity in their spectra to the reference strains of *B. longum* subsp. *longum* in the Biotyper database, indicating that the strains labelled as *B. longum* all belonged to the subspecies *longum* and that the MALDI-TOF MS analysis could distinguish between these two subspecies. In general, PCR identification still offers advantage over MALDI-TOF MS analysis in the ability of accurate identification down to subspecies level, which was also evident in this study, i.e., in the case of *Streptococcus salivarius* subsp. *thermophilus*, *B. animalis* subsp. *lactis*, *B. longum* subsp. *longum*, and *B. longum* subsp. *infantis*.

Another problem observed in this study concerned the representatives of *L. casei* and *L. paracasei* in the products, as some colonies were identified as *L. paracasei* or *L. zeae*, although the latter species was not indicated on the labels. Misidentification of *L. casei* and related species has been reported previously (Morovic et al., 2016; Patro et al., 2016). A recent study showed that multiplex PCR and MALDI-TOF MS proved to be the most useful methods for species-level identification of bacteria belonging to the *L. casei* group (Jarocki et al., 2020).

The species *L. zeae*, which was one of the frequent matches in the analyses of colonies by MALDI-TOF MS, was not found among the bacteria listed on the labels of the products examined in this study. It is well known that *L. casei*, *L. paracasei*, and *L. rhamnosus*, species collectively referred to as the *Lactobacillus casei* group, are difficult to distinguish from each other and have been reclassified several times in the past (Hill et al., 2018). In 2008, the type strain of *L. casei* was confirmed as the original strain ATCC 393, and strain ATCC 334 was designated as *L. paracasei*. The species name *L. zeae* was reclassified as *L. casei* in 2008 (Tindall and Syste, 2008) and reassigned to distinct species status in 2020 (Liu and Gu, 2020). Therefore, the confusion around the species *L. zeae*, which is found in the Biotyper database but absent from the labels, is not surprising. Moreover, there are only two *L. zeae* strains and one *L. casei* strain in the Biotyper 1.3.66 database compared to 13 *L. rhamnosus* strains and 15 *L. paracasei* strains. Another fact that complicates the routine identification of *L. zeae* and closely related species is that PCR analyses based on 16S rDNA, as the most common taxonomic marker, are not always reliable due to the high sequence similarities between the three species (*L. casei*, *L. rhamnosus*, and *L. zeae*). Since many strains within the *L. casei* group are commonly used as starter cultures and probiotics, the possibility of improper labelling should be considered. In this study, colonies that showed similar MALDI-TOF MS spectra to *L. zeae*, but with lower score values (1.700–1.999), were identified as *L. rhamnosus* by using species-specific primers designed not to amplify DNA from *L. zeae* and *L. casei* (Walter et al., 2000) and also using primers from Bottari et al. (2017) that can distinguish between *L. casei*, *L. rhamnosus*, and *L. paracasei*. In addition, the affiliation to this species was confirmed also by 16S rDNA sequencing.

At least one strain, *L. casei* W56, in two products examined in this study (2, 12) was found to be mislabelled since the species *L. casei* in these products could not be confirmed by either species-specific PCR or MALDI-TOF MS, while the species *L. paracasei* was detected in both products. This is in line with the fact that the strain W56 is already available in GenBank (accession number HE970765.1) and indeed belongs to the species *L. paracasei*. Similar observations were reported by Kim et al. (2020) who examined 29 products containing members of the *L. casei* group using newly designed PCR primers based on the comparative genetics of whole-genome sequencing (Kim et al., 2020). They found that 23 products purchased in markets around the world (Korea, Canada, and the United States of America) were consistent with label claims, while the remaining products contained species different from those indicated in the label claims. They also confirmed that the 16S rRNA gene sequence was inadequate for distinguishing between species in the *L. casei* group. The misidentification of a commercial *L. casei* strain (DG, CNCM I-1572) found in an Italian pharmaceutical product was also reported by Blandino et al. (2016), who successfully used multiplex PCR based on tuf gene amplification (Blandino et al., 2016).

The absence of certain strains was observed in most of the products for general use (11 out of 17), all of which contained multiple strains, and only in one product for children (25). This problem has been previously reported in several studies based on PCR assessment, high-throughput next-generation sequencing, or denaturing gradient gel electrophoresis analysis (Morovic et al., 2016; Patro et al., 2016; Chen et al., 2017; Shehata and Newmaster, 2020). For 10% of the labelled strains (11 of 112 examined), presence was confirmed by species-specific PCR with viable consortia, but not by MALDI-TOF MS analysis of individual colonies, with three colonies per labelled species included in the analysis. This could be improved by analysing more colonies using MALDI-TOF MS analysis.

The absence of positive results with viable consortia or colonies, but positive results with DNA by PCR using DNA isolated directly from the products observed in 10% (11/112) of the labelled strains, indicates the presence of non-culturable bacteria in the products. However, the labelled bacteria are expected to be in a viable and culturable state since the amount of bacteria is expressed in CFU/g or CFU/unit of product. This is the main reason why quantitative PCR or other molecular methods cannot easily replace cultivation-based methods (Kramer et al., 2009).

Identification of LAB and bifidobacteria in probiotic products and starter cultures using MALDI-TOF MS has not been as widely used in the past, mainly because of the high cost of the equipment. Moreover, the identification of LAB by MALDI-TOF MS was not very satisfactory at the beginning because of the lack of high-quality databases containing the spectra of several reference strains of this group of bacteria. Nowadays, commercial databases such as the MALDI Biotyper (Bruker Daltonics), the SARAMIS^TM^ (BioMerieux), and the Andromas (Andromas SAS), as well as other open-source databases that are regularly updated, are available, which allows better discrimination between closely related species (Čanžek Majhenič et al., 2018). In the present study, the Biotyper database allowed the satisfactory identification of most species present in dietary supplements, with some exceptions such as *L. zeae* and *L. casei*, as well as those that could not be accurately distinguished by spectra comparisons. *L. paracasei* and *L. rhamnosus*, on the other hand, could be distinguished from *L. casei*, although the species are closely related and difficult to distinguish based on 16S rDNA sequences. For example, Huang and Huang (2018) validated the accuracy of MALDI-TOF MS in identifying three related species belonging to the genus *Lacticaseibacillus*, i.e., *L. casei*, *L. paracasei*, and *L. rhamnosus*, and concluded that the in-house database containing the spectra of several reference strains grown under optimised culture conditions, in combination with ClinProTools, can improve identification by MALDI-TOF MS and enable the identification of subspecies (*L. paracasei* subsp. *paracasei* and *L. paracasei* subsp. *tolerans*) (Huang and Huang, 2018). They were able to detect *L. paracasei* in two yoghurt drinks, *L. casei* in two probiotic preparations, and *L. rhamnosus* in three probiotic preparations. *L. zeae*, on the other hand, was not included in their study.

An interesting observation was that, even in the products containing only one strain, the probability of correct MALDI-TOF MS identification of *B. longum* varied among three colonies, i.e., one or two were identified to species level with score values indicating probable species identification, while another one or two showed only probable genus identification (score value between 1.700 and 1.999). The reason for this discrepancy can be found in the fact that the quality of the spectrum may be influenced by several factors, including the amount of bacteria spotted on the target and, in the case of anaerobic bacteria, the exposure of the bacterial cells to oxygen. Veloo et al. (2014) reported that *B. longum* required on-target extraction with 70% formic acid to obtain reliable species identification and that the quality of the spectrum was influenced by the amount of bacteria, the homogeneity of the smear, and the experience of the investigator (Veloo et al., 2014). As a too-low amount of bacteria can result in the absence of a peak, a too-high amount can result in an atypical spectrum due to saturation and the predominance of prominent peaks.

In our study, 30% of isolates were identified with score values ranging from 1.700 to 1.999, values considered too low for accurate species identification using the manufacturer’s cutoff values. Nevertheless, species affiliation was confirmed in almost all cases by 16S rDNA sequencing and/or PCR, suggesting that the general criteria for the interpretation of MALDI-TOF MS results may be too stringent in the case of LAB and bifidobacteria. In a study by Gautam et al. (2017), MALDI-TOF MS-based identification of non-fermenting Gram-negative bacilli at the species level was successful when the cutoff value was decreased. A 4% increase in species identification rate was achieved when the cutoff of ≥1.9 was taken instead of ≥2.

Several other studies have shown that score values and identification accuracy can be significantly improved by using in-house-built reference database containing several MALDI-TOF MS spectra of reference strains and own isolates of interest, depending on the sample composition and scope of the analyses, and by using a more advanced analysis of the spectra (Singhal et al., 2015; Nacef et al., 2017; Gantzias et al., 2020; Jarocki et al., 2020). However, it should be kept in mind that, in this study, we intentionally used only the commercially available Biotyper library to demonstrate whether the MALDI-TOF MS approach is widely applicable in high-throughput routine laboratories for the control of probiotic products, without extensive expertise in this methodology.

## Conclusion

In conclusion, this study showed a good quality of most products (88%) in terms of total CFU/g. Considering the results of our study, we can also conclude that MALDI-TOF MS could not accurately distinguish between *L. zeae* and *L. casei*, while *L. paracasei* and *L. rhamnosus* could be correctly identified. Some isolates that were identified as *L. zeae* by MALDI-TOF MS but had lower score values (1.700–1.999), indicating unreliable species identification, were most likely *L. rhamnosus* as indicated by species-specific PCR analysis. Moreover, all these isolates were obtained from the product containing *L. rhamnosus* on the labels. The inability of identification of *L. acidophilus* La-5 and *B. coagulans* with MALDI-TOF MS could be explained by the low number of reference spectra (three and two, respectively) for these two species in the Biotyper database or by the low-quality spectra obtained. Otherwise, e.g., 15 fingerprints of *L. paracasei*, 13 of *L. rhamnosus*, and 9 of *L. gasseri* are available for comparison.

PCR is still considered the more reliable of MALDI-TOF MS, especially for subspecies or strain-level identification, but compared to MALDI-TOF MS, PCR analysis is more labour intensive and requires more trained personnel. MALDI-TOF MS is easier to use, faster, cheaper, and allows testing of a large number of colonies if needed (in case of negative results for a particular species). Nevertheless, our results confirm that MALDI-TOF MS can be effectively used in the quality control of probiotic products considering some limitations, such as unsatisfactory identification of certain closely related species. In such cases, PCR confirmation can help. Furthermore, generation of a dedicated in-house library containing MALDI-TOF MS spectra of various reference strains and isolates of interest may further improve the accuracy of identification at the species and sub-species level.

## Data Availability Statement

The original contributions presented in the study are included in the article/Supplementary Material, further inquiries can be directed to the corresponding author/s.

## Author Contributions

PM, BB, and MK designed the experiments. PM and MG performed the experiments and collected the data. PM, PT, and BB conducted all analyses. PM, BB, PT, and MK prepared the manuscript. All the authors have contributed to, seen, and approved the manuscript.

## Conflict of Interest

MK was employed by company Lek Pharmaceuticals d.d. The remaining authors declare that the research was conducted in the absence of any commercial or financial relationships that could be construed as a potential conflict of interest.

## Footnotes

1 http://blast.ncbi.nlm.nih.gov/blast/

## Supplementary Material

Supplementary Table 1 Species-specific PCR primers and conditions.

Supplementary Table 2 Compliance of 26 dietary supplements with the labelled number of colony-forming units.

Supplementary Table 3 Results of the identification of isolates from nine dietary supplements for children (C1–A9) by MALDI-TOF MS analysis and by species-specific PCR (three isolates per labelled strain).

Supplementary Table 4 Overview of the MALDI-TOF MS (Biotyper) identification of 70 isolates (three isolates per labelled strain) from 9 dietary supplements for children (products 18–26).

## Abbreviations

*B.*: *Bifidobacterium*
*Bac.*: *Bacillus*
*E.*: *Enterococcus*
*L.*: former *Lactobacillus* genus now comprising new genera (*Lactobacillus, Lacticaseibacillus, Limosilactobacillus, Lactiplantibacillus*, and *Ligilactobacillus*)
*Lc.*: *Lactococcus*
MALDI-TOF MS: matrix-assisted laser desorption/ionisation time-of-flight mass spectrometry
*Sacch.*: *Saccharomyces*.
