# Supplementary material for: Evaluation of Dietary Supplements Containing Viable Bacteria by Cultivation/MALDI-TOF Mass Spectrometry and PCR Identification
Source: Front Microbiol. 2021 Jul 19;12:700138. doi: 10.3389/fmicb.2021.700138 (PMC8326757; doi:10.3389/fmicb.2021.700138)
Supplement: Supplementary Table 1 — Species-specific PCR primers and conditions. [file Data_Sheet_1.docx]

Supplementary Material

**Supplementary Table 1.** Species-specific PCR primers and conditions

| Target species | Primers | | PCR protocol | N^o^ of  cycles | Product length | References |
| --- | --- | --- | --- | --- | --- | --- |
| *Bacillus coagulans* | | B.coag-F: 5'-TACGGCATTGGCAAGTATCA-3'  B.coag-R: 5'-CGACATGATTTGGTTTTCCA-3' | 95 °C/5 min | 1 | 555 bp | Liu et al., 2010 |
|  |  |  | 95 °C/30 s  56 °C/30 s  72 °C/30 s | 30 |  |  |
|  |  |  | 72 °C/10 min | 1 |  |  |
| *Bifidobacterium animalis* | | B_ani-f: 5'-CACCAATGCGGAAGACCAG-3'  B_ani-r: 5'-GTTGTTGAGAATCAGCGTGG-3' | 95 °C/3 min | 1 | 184 bp | Junick and Blaut, 2012 |
|  |  |  | 95 °C/15 s  62 °C/20 s  72 °C/30 s | 30 |  |  |
|  |  |  | 72 °C/5 min | 1 |  |  |
| *Bifidobacterium animalis* subsp. *lactis* | | BflactF: 5'-CCCTTTCCACGGGTCCC-3'  BflactR: 5'-AAGGGAAACCGTGTCTCCAC-3' | 95 °C/3 min | 1 | 194 bp | Malinen et al., 2003 |
|  |  |  | 95 °C/15 s  62 °C/20 s  72 °C/30 s | 30 |  |  |
|  |  |  | 72 °C/5 min | 1 |  |  |
| *Bifidobacterium bifidum* | | BiBIF-1: 5'- CCACATGATCGCATGTGATTG -3'  BiBIF-2: 5'- CCGAAGGCTTGCTCCCAAA -3' | 95 °C/3 min | 1 | 278 bp | Matsuki et al., 1999 |
|  |  |  | 95 °C/30 s  55 °C/30 s  72 °C/30 s | 30 |  |  |
|  |  |  | 72 °C/5 min | 1 |  |  |
| *Bifidobacterium breve* | | IDB31F: 5'-TAGGGAGCAAGGCACTTTGTGT-3'  IDBC1R: 5'-ATCCGAACTGAGACCGGTT-3' | 95 °C/3 min | 1 | 827 bp | Kwon et al., 2005 |
|  |  |  | 95 °C/30 s  64 °C/30 s  72 °C/1 min | 30 |  |  |
|  |  |  | 72 °C/5 min | 1 |  |  |
| *Bifidobacterium longum* | | F: 5'-CAGTTGATCGCATGGTCTT-3'  R: 5'- TACCCGTCGAAGCCAC -3' | 95 °C/3 min | 1 | 106 bp | Malinen et al., 2003 |
|  |  |  | 95 °C/15 s  60 °C/20 s  72 °C/30 s | 35 |  |  |
|  |  |  | 72 °C/5 min | 1 |  |  |
| *Bifidobacterium longum* subsp*. infantis* | | BiINF-1: 5'-TTCCAGTTGATCGCATGGTC-3'  BiINF-2: 5'-GGAAACCCCATCTCTGGGAT-3' | 95 °C/3 min | 1 |  | Matsuki et al., 1999 |
|  |  |  | 95 °C/30 s  55 °C/30 s  72 °C/30 s | 35 | 828 bp |  |
|  |  |  | 72 °C/5 min | 1 |  |  |
| *Enterococcus faecium* | | ddl F1: 5'- GCAAGGCTTCTTAGAGA -3'  ddl F2: 5'- CATCGTGTAAGCTAACTTC -3' | 95 °C/3 min | 1 |  | Dutka-Malen  et al., 1995 |
|  |  |  | 95 °C/30 s  55 °C/30 s  72 °C/30 s | 30 | 550 bp |  |
|  |  |  | 72 °C/5 min | 1 |  |  |
| *Lactobacillus acidophilus* | | LacI: 5'-AGCTGAACCAACAGATTCAC-3'  LacII: 5'-ACTACCAGGGTATCTAATCC-3' | 95 °C/3 min | 1 | 759 bp | Walter et al., 2000 |
|  |  |  | 95 °C/30 s  62 °C/30 s  72 °C/30 s | 30 |  |  |
|  |  |  | 72 °C/5 min | 1 |  |  |
| *Lactocaseibacillus casei* | | Cas1: 5'-TGCACTGAGATTCGACTTAA-3'  Y2: 5'-CCCACTGCTGCCTCCCGTAGGAGT-3' | 95 °C/3 min | 1 | ~300 bp | Ward et al., 1999 |
|  |  |  | 95 °C/30 s  55 °C/30 s  72 °C/30 s | 30 |  |  |
|  |  |  | 72 °C/5 min | 5 |  |  |
| *Lb. delbrueckii* subsp*. bulgaricus* | | LB1: 5'-AAAAATGAAGTTGTTTAAAGTAGGTA-3'  LLB1: 5'-AAGTCTGTCCTCTGGCTGG-3' | 95 °C/3 min | 1 | 1065 bp | Torriani et al., 1999 |
|  |  |  | 95 °C/30 s  58 °C/30 s  72 °C/30 s | 30 |  |  |
|  |  |  | 72 °C/5 min | 1 |  |  |
| *Limosilactobacillus fermentum* | | Lfpr: 5'-GCCGCCTAAGGTGGGACAGAT-3'  FermІІ: 5'-CTGATCGTAGATCAGTCAAG-3' | 95 °C/3 min | 1 | 200-300 bp | Walter et al.,  2000 |
|  |  |  | 95 °C/30 s  58 °C/30 s  72 °C/30 s | 30 |  |  |
|  |  |  | 72 °C/5 min | 1 |  |  |
| *Lactobacillus. gasseri* | | Lgas2: 5'-TGCTATCGCTTCAAGTGCTT-3'  Lgas3: 5'-AGCGACCGAGAAGAGAGAGA-3' | 95 °C/3 min | 1 |  | Song et al., 2000 |
|  |  |  | 95 °C/30 s  55 °C/30 s  72 °C/30 s |  | 360 bp |  |
|  |  |  | 72 °C/5 min | 1 |  |  |
| *Lactocaseibacillus paracasei* | | Para1: 5'-CACCGAGATTCAACATGG-3'  Y2: 5'-CCCACTGCTGCCTCCCGTAGGAGT-3' | 95 °C/3 min | 1 | 290 bp | Ward et al., 1999 |
|  |  |  | 95 °C/30 s  55 °C/30 s  72 °C/30 s | 30 |  |  |
|  |  |  | 72 °C/5 min | 1 |  |  |
| *Lactiplantibacillus plantarum* | | Plant 1: 5'-ATCATGATTTACATTTGAGTG-3'  LOWLAC: 5'- CGACGACCATGAACCACCTGT -3' | 95 °C/3 min | 1 |  | Chagnaud in  sod., 2001 |
|  |  |  | 95 °C/30 s  58 °C/30 s  72 °C/30 s | 30 | 996 bp |  |
|  |  |  | 72 °C/5 min | 1 |  |  |
| *Limosilactobacillus reuteri* | | Lfpr: 5'- GCCGCCTAAGGTGGGACAGAT -3'  Reu: 5'- AACACTCAAGGATTGTCTGA -3' | 95 °C/2 min | 1 |  | Walter et al., 2000 |
|  |  |  | 95 °C/30 s  55 °C/30 s  72 °C/30 s | 30 | 200-300 bp |  |
|  |  |  | 72 °C/5 min | 1 |  |  |
| *Lactocaseibacillus rhamnosus* | | PrI: 5'- CAGACTGAAAGTCTGACGG -3'  RhaII: 5'- GCGATGCGAATTTCTATTATT -3' | 95 °C/3 min | 1 | 186 bp | Walter et al., 2000 |
|  |  |  | 95 °C/30 s  58 °C/30 s  72 °C/30 s | 30 |  |  |
|  |  |  | 72 °C/5 min | 1 |  |  |
| *Ligilactobacillus salivarius* | | Sal1: 5'-ATTCACTCGTAAGAAGT-3'  LOWLAC: 5'-CGACGACCATGAACCACCTGT-3' | 95 °C/3 min | 1 |  | Chagnaud in  sod., 2001 |
|  |  |  | 95 °C/30 s  50 °C/30 s  72 °C/1 min | 30 | 993 bp |  |
|  |  |  | 72 °C/5 min | 1 |  |  |
| *Lactococcus lactis* | | 27f: 5'- AGAGTTTGATCMTGGCTCAG -3'  LIa: 5'- CAGTCGGTACAAGTACCAAC -3' | 95 °C/2 min | 1 | 87 bp | Barakat et al., 2000 |
|  |  |  | 95 °C/30 s  55 °C/30 s  72 °C/30 s | 30 |  |  |
|  |  |  | 72 °C/5 min | 1 |  |  |
| *Saccharomyces cerevisiae* | | SC1: 5'-AACGGTGAGAGATTTCTGTGC-3'  SC2: 5'-AGCTGGCAGTATTCCCACAG-3' | 95 °C/3 min | 1 | 1170 bp | Josepa et al., 2000 |
|  |  |  | 95 °C/30 s  50 °C/30 s  72 °C/1 min | 30 |  |  |
|  |  |  | 72 °C/5 min | 1 |  |  |
| *Streptococcus salivarius* subsp. *thermophilus* | | ThI: 5'-ACGGAATGTACTTGAGTTTC-3'  ThII: 5'-TTTGGCCTTTCGACCTAAC-3' | 95 °C/3 min | 1 | 205-304 bp | Tilsala-Timisjärvi in Alatossava, 1997 |
|  |  |  | 95 °C/30 s  58 °C/30 s  72 °C/30 s | 30 |  |  |
|  |  |  | 72 °C/5 min | 1 |  |  |

**Supplementary Table 2.** Compliance of 26 dietary supplements with the labelled number of CFU.

|  | **Labelled strains** | **Time till the end of shelf life (months)** | **Labelled number (CFU)** | **Number (CFU) determined by plate counting** | **Compliance of CFU number with the label** |  |
| --- | --- | --- | --- | --- | --- | --- |
|  | **Dietary supplements for adults** | | | | | |
| 1 | *L. acidophilus* La-14, *L. plantarum* Lp-115, *L. rhamnosus* Lr-32, *B. breve* BB-03, *L. salivarius* Ls-33, *B. lactis* Bl-04, *L. casei* LC-11, *L. paracasei* Lpc-37, *S. thermophilus* St-21, *B. longum* Bl-05 | 13 | 2.5×10^10^ CFU/capsule | 1.1×10^11^ CFU/g  3.9×10^10^ CFU/capsule | Yes |  |
| 2 | *L. casei* W56, *L. acidophilus* W22, *L. paracasei* W20, *B. lactis* W51, *L. salivarius* W24, *Lc. lactis* W19, *B. lactis* W52, *L. plantarum* W62, *B. bifidum* W23 | 12 | 7.5×10^9^/sachet | 5.4×10^9^ CFU/g  1.6×10^10^ CFU/sachet | Yes |  |
| 3 | *L. acidophilus, L. casei, L. plantarum, L. reuteri, L. rhamnosus, B. longum, S. thermophilus* | 14 | 1×10^9^ CFU/tablet | 3.2×10^8^ CFU/g  1.3×10^8^ CFU/tablet | Too low number of CFU  (for 0.89 log_10_) |  |
| 4 | *B. animalis* subsp. *lactis* A026, *L. paracasei* A234, *B. breve* A055, *L. gasseri* A237, *L. rhamnosus* A 119, *L. rhamnosus* A 11993, *L. acidophilus* A118, *L. plantarum* A138, *B. longum* subsp. *longum* A027, *B. bifidum* A 058, *L. casei* A 179, *L. reuteri* A 113, *Lc. lactis* A 328, *B. longum* subsp. *infantis*  A041 | 16 | 1×10^10^ CFU/capsule | 8.4×10^10^ CFU/g  4.2×10^10^ CFU/capsule | Yes |  |
| 5 | *L. acidophilus* La-14, *B. lactis* Bl-04, *L. plantarum* Lp-115, *B. breve* Bb-03 | 15 | 4×10^9^ CFU/capsule | 4.9×10^9^ CFU/g  1.5×10^9^ CFU/capsule | Partially  (lower for 0.43 log_10_) |  |
| 6 | *Bac. coagulans* | 14 | 5×10^9^/g  2×10^9^/capsule | 2.3×10^9^ CFU/g  9.2×10^8^ CFU/capsule | Partially  (lower for 0.34 log_10_) |  |
| 7 | *L. acidophilus* DDS-1, *B. lactis,*  *L. plantarum, L. rhamnosus, L. casei, B. longum, S. thermophilus* | 12 | 2×10^10^/capsule | 1.4×10^10^ CFU/g  2.8×10^9^ CFU/capsule | Too low number of CFU  (for 0.85 log_10_) |  |
| 8 | *S. thermophilus* DSM 24731, *B. breve* DSM 24732, *B. longum* DSM 2473, *B. infantis* DSM 24737, *L. acidophilus* DSM 24735, *L. plantarum* DSM 24730, *L. paracasei* DSM 24733, *L. delbrueckii* subsp. *bulgaricus* DSM 24734 | 12 | 4.5×10^11^ CFU/sachet | 1.1×10^11^ CFU/g  4.8×10^11^ CFU /sachet | Yes |  |
| 9 | *L. rhamnosus* GG (ATCC 53103) | 14 | 1×10^10^ CFU/capsule | 3.0×10^9^ CFU/g  1.5×10^9^ CFU/capsule | Too low number of CFU  (for 0.82 log) |  |
| 10 | *L. reuteri Protectis* (DSM 17938) | 11 | 1×10^9^ CFU/tablet | 2.4×10^9^ CFU/g  1.1×10^9^ CFU/tablet | Yes |  |
| 11 | *Sacch. boulardii*, *L. acidophilus, B. breve, B. infantis, B. longum* | 19 | Yeasts:5×10^9^ CFU/capsule  Bacteria:1.5×10^9^ CFU/capsule | Yeasts:  6.5×10^9^ CFU/g  3.6×10^9^ CFU/capsule  Bacteria:  1.7×10^10^ CFU/g  9.5×10^9^ CFU/capsule | Yeasts: partially  (lower for 0.14 log)  Bacteria: Yes |  |
| 12 | *B. animalis* W53, *L. acidophilus* W55, *L. salivarius* W57, *E. faecium* W54, *Lc. lactis* W58, *L. casei* W56 | 28 | 3×10^9^/sachet | 2.8×10^9^ CFU/g  8.4×10^9^ CFU/sachet | Yes |  |
| 13 | *L. acidophilus* W55, *L. acidophilus* W37, *L. paracasei* W72, *L. rhamnosus* W71, *E. faecium* W54, *L. salivarius* W24, *L. plantarum* W62, *B. bifidum* W23, *B. lactis* W18, *B. longum* W51 | 10 | 8×10^9^/sachet | 2.0×10^9^ CFU/g  1.0×10^10^ CFU/sachet | Yes |  |
| 14 | *L. rhamnosus* GG | 16 | 1.2×10^10^ CFU/capsule | 2.2×10^11^ CFU/g  3.7×10^10^ CFU/capsule | Yes |  |
| 15 | *L. acidophilus* (*L. gasseri* species), *B. infantis, E. faecium* | 5 | 1.2×10^7^ CFU/capsule | 2.2×10^8^ CFU/g  6.2×10^7^ CFU/capsule | Yes |  |
| 16 | *L. acidophilus* LA-5, *B. animalis* subsp. *lactis* BB-12 | 20 | 2×10^9^ CFU/capsule | 3.2×10^10^ CFU/g  7.7×10^9^ CFU/capsule | Yes |  |
| 17 | *L. plantarum, L. fermentum, L. acidophilus, L. reuteri, L. rhamnosus, B. bifidum* | 19 | 1.8×10^9^ CFU/capsule | 4.6×10^10^ CFU/g  2.5×10^10^ CFU/capsule | Yes |  |
|  | **Dietary supplements for children** | | | | | |
| 18 | *B. animalis* subsp*. lactis* (BB-12) | 11 | 1×10^9^ CFU/sachet | 6.8 × 10^9^/sachet | Yes |  |
| 19 | *B. animalis* subsp*. lactis* (BB-12) | 2 | 1×10^9^ CFU/6 drops | 1.4 × 10^10^/6 drops | Yes |  |
| 20 | *Lb. reuteri* DSM 17938 *(L. reuteri* protectis*)* | 11 | 10^8^ viable bacteria/5 drops | 4.3 × 10^8^/5 drops | Yes |  |
| 21 | *Lb. reuteri* DSM 17938 *(Lb. reuteri* Protectis*)* | 19 | 10^8^ viable bacteria/5 drops | 5.1 × 10^8^/5 drops | Yes |  |
| 22 | *Lb. rhamnosus* GG (ATCC 53103) | 16 | 1.4×10^9^ CFU/7 drops | 8.4 × 10^8^/7 drops | Partially  (lower for 0.22 log_10_) |  |
| 23 | *Lb. rhamnosus* GG (ATCC 53103) | 16 | 1.4×10^9^ CFU /7 drops | 8.7 × 10^8^/7 drops | Partially  (lower for 0.21 log_10_) |  |
| 24 | *Lb. rhamnous* (LGG), *B. animalis* subsp. *lactis* (BB-12) | 2 | 10^9^ viable bacteria/6 drops | 1.1 × 10^10^/6 drops | Yes |  |
| 25 | *Lb. casei* (NCIMB 30185) PXN 37, *Lb. rhamnosus* (NCIMB 30188) PXN 54, *S. thermophilus* (NCIMB 30189) PXN 66, *B. breve* (NCIMB 30180) PXN 25, *Lb. acidophilus* (NCIMB 30184) PXN 35, *B. infantis* (NCIMB 30181) PXN 27, *Lb. bulgaricus* (NCIMB 30186) PXN 39 | 14 | 1×10^9^ CFU/sachet | 9,4 × 10^9^/sachet | Yes |  |
| 26 | *Lc. lactis* W58, *B. lactis* W52, *B. bifidum* W23 | 19 | 3×10^9^ CFU/sachet | 1.3 × 10^10^/sachet | Yes |  |

CFU – colony forming units; *Bac.. Bacillus; B.. Bifidobacterium; B. lactis, Bifidobacterium animalis* subsp. *lactis; B. infantis, B. longum* subsp. *infantis; E.. Enterococcus; L.. Lactobacillus* (*in accordance with new taxonomic classification.* Zheng *et al.* (2020). also *Lactocaseibacillus. Lactiplantibacillus. Limosilactobacillus. Ligilactobacillus*); *Lb. bulgaricus,* *Lb. delbrueckii* subsp*. bulgaricus; Lc.. Lactococcus; S. thermophilus, Streptococcus salivarius* subsp. *thermophilus;*

**Supplementary Table 3:** Results of the identification of isolates from 17 dietary supplemens (1-17) by MALDI-TOF MS analysis and by species specific PCR (3 isolates per labelled strain).

_________________________________________________________________­­­­­­­­­­­­­­­­­

| **Product** | **Isolate designation** | **Biotyper 3.1 result**  **(best match)** | **ScoreValue** | **PCR individual colonies** | **16S rDNA sequencing** |  |
| --- | --- | --- | --- | --- | --- | --- |
| 1 | 1a | *Streptococcus salivarius_ssp_thermophilus* | [2.164](file:///D:\Diplome\Jo%C5%BEi%20Omahen-probioti%C4%8Dni%20izdelki%20Lek\Maldi%20rezultati\Bruker%20Daltonik%20MALDI%20Biotyper%20Classification%20Results.htm#ID0EA) | *S. thermophilus* | *n.d.* |  |
|  | 1b | *Streptococcus salivarius_ssp_thermophilus* | [2.033](file:///D:\Diplome\Jo%C5%BEi%20Omahen-probioti%C4%8Dni%20izdelki%20Lek\Maldi%20rezultati\Bruker%20Daltonik%20MALDI%20Biotyper%20Classification%20Results.htm#ID0EA) | *S. thermophilus* | *n.d.* | |
|  | 1c | *Streptococcus salivarius_ssp_thermophilus* | [1.854](file:///D:\Diplome\Jo%C5%BEi%20Omahen-probioti%C4%8Dni%20izdelki%20Lek\Maldi%20rezultati\Bruker%20Daltonik%20MALDI%20Biotyper%20Classification%20Results.htm#ID0EA) | *S. thermophilus* | *n.d.* | |
|  | 1d | [*Lactobacillus acidophilus*](file:///D:\Diplome\Jo%C5%BEi%20Omahen-probioti%C4%8Dni%20izdelki%20Lek\Maldi%20rezultati\Bruker%20Daltonik%20MALDI%20Biotyper%20Classification%20Results.htm#ID0EOCA) | [2.411](file:///D:\Diplome\Jo%C5%BEi%20Omahen-probioti%C4%8Dni%20izdelki%20Lek\Maldi%20rezultati\Bruker%20Daltonik%20MALDI%20Biotyper%20Classification%20Results.htm#ID0EA) | *L. acidophilus* | *n.d.* | |
|  | 1e | [*Lactobacillus acidophilus*](file:///D:\Diplome\Jo%C5%BEi%20Omahen-probioti%C4%8Dni%20izdelki%20Lek\Maldi%20rezultati\Bruker%20Daltonik%20MALDI%20Biotyper%20Classification%20Results.htm#ID0EOCA) | [2.359](file:///D:\Diplome\Jo%C5%BEi%20Omahen-probioti%C4%8Dni%20izdelki%20Lek\Maldi%20rezultati\Bruker%20Daltonik%20MALDI%20Biotyper%20Classification%20Results.htm#ID0EA) | *L. acidophilus* | *n.d.* | |
|  | 1f | *Lactobacillus plantarum* | [2.162](file:///D:\Diplome\Jo%C5%BEi%20Omahen-probioti%C4%8Dni%20izdelki%20Lek\Maldi%20rezultati\Bruker%20Daltonik%20MALDI%20Biotyper%20Classification%20Results.htm#ID0EA) | *L. plantarum* | *n.d.* | |
|  | 1g | [*Lactobacillus acidophilus*](file:///D:\Diplome\Jo%C5%BEi%20Omahen-probioti%C4%8Dni%20izdelki%20Lek\Maldi%20rezultati\Bruker%20Daltonik%20MALDI%20Biotyper%20Classification%20Results.htm#ID0EOCA) | [2.467](file:///D:\Diplome\Jo%C5%BEi%20Omahen-probioti%C4%8Dni%20izdelki%20Lek\Maldi%20rezultati\Bruker%20Daltonik%20MALDI%20Biotyper%20Classification%20Results.htm#ID0EA) | *L. acidophilus* | *n.d.* | |
|  | 1h | [*Lactobacillus acidophilus*](file:///D:\Diplome\Jo%C5%BEi%20Omahen-probioti%C4%8Dni%20izdelki%20Lek\Maldi%20rezultati\Bruker%20Daltonik%20MALDI%20Biotyper%20Classification%20Results.htm#ID0EOCA) | [2.197](file:///D:\Diplome\Jo%C5%BEi%20Omahen-probioti%C4%8Dni%20izdelki%20Lek\Maldi%20rezultati\Bruker%20Daltonik%20MALDI%20Biotyper%20Classification%20Results.htm#ID0EA) | *L. acidophilus* | *n.d.* | |
|  | 1i | [*Lactobacillus acidophilus*](file:///D:\Diplome\Jo%C5%BEi%20Omahen-probioti%C4%8Dni%20izdelki%20Lek\Maldi%20rezultati\Bruker%20Daltonik%20MALDI%20Biotyper%20Classification%20Results.htm#ID0EOCA) | [2.364](file:///D:\Diplome\Jo%C5%BEi%20Omahen-probioti%C4%8Dni%20izdelki%20Lek\Maldi%20rezultati\Bruker%20Daltonik%20MALDI%20Biotyper%20Classification%20Results.htm#ID0EA) | *L. acidophilus* | *n.d.* | |
|  | 1j | *Lactobacillus plantarum* | [1.988](file:///D:\Diplome\Jo%C5%BEi%20Omahen-probioti%C4%8Dni%20izdelki%20Lek\Maldi%20rezultati\Bruker%20Daltonik%20MALDI%20Biotyper%20Classification%20Results.htm#ID0EA) | *L. plantarum* | *n.d.* | |
|  | 1k | *Lactobacillus plantarum* | [1.809](file:///D:\Diplome\Jo%C5%BEi%20Omahen-probioti%C4%8Dni%20izdelki%20Lek\Maldi%20rezultati\Bruker%20Daltonik%20MALDI%20Biotyper%20Classification%20Results.htm#ID0EA) | *L. plantarum* | *n.d.* | |
|  | 1l | [*Lactobacillus acidophilus*](file:///D:\Diplome\Jo%C5%BEi%20Omahen-probioti%C4%8Dni%20izdelki%20Lek\Maldi%20rezultati\Bruker%20Daltonik%20MALDI%20Biotyper%20Classification%20Results.htm#ID0EOCA) | [2.388](file:///D:\Diplome\Jo%C5%BEi%20Omahen-probioti%C4%8Dni%20izdelki%20Lek\Maldi%20rezultati\Bruker%20Daltonik%20MALDI%20Biotyper%20Classification%20Results.htm#ID0EA) | *L. acidophilus* | *n.d.* | |
|  | 1m | [*Lactobacillus acidophilus*](file:///D:\Diplome\Jo%C5%BEi%20Omahen-probioti%C4%8Dni%20izdelki%20Lek\Maldi%20rezultati\Bruker%20Daltonik%20MALDI%20Biotyper%20Classification%20Results.htm#ID0EOCA) | [2.449](file:///D:\Diplome\Jo%C5%BEi%20Omahen-probioti%C4%8Dni%20izdelki%20Lek\Maldi%20rezultati\Bruker%20Daltonik%20MALDI%20Biotyper%20Classification%20Results.htm#ID0EA) | *L. acidophilus* | *n.d.* | |
|  | 1n | *Lactobacillus plantarum* | [1.782](file:///D:\Diplome\Jo%C5%BEi%20Omahen-probioti%C4%8Dni%20izdelki%20Lek\Maldi%20rezultati\Bruker%20Daltonik%20MALDI%20Biotyper%20Classification%20Results.htm#ID0EA) | *L. plantarum* | *n.d.* | |
|  | 1o | [*Lactobacillus acidophilus*](file:///D:\Diplome\Jo%C5%BEi%20Omahen-probioti%C4%8Dni%20izdelki%20Lek\Maldi%20rezultati\Bruker%20Daltonik%20MALDI%20Biotyper%20Classification%20Results.htm#ID0EOCA) | [2.378](file:///D:\Diplome\Jo%C5%BEi%20Omahen-probioti%C4%8Dni%20izdelki%20Lek\Maldi%20rezultati\Bruker%20Daltonik%20MALDI%20Biotyper%20Classification%20Results.htm#ID0EA) | *L. acidophilus* | *n.d.* | |
|  | 1p | ***no peaks found*** | [< 0](file:///D:\Diplome\Jo%C5%BEi%20Omahen-probioti%C4%8Dni%20izdelki%20Lek\Maldi%20rezultati\Bruker%20Daltonik%20MALDI%20Biotyper%20Classification%20Results.htm#ID0EA) | *L. acidophilus* | *n.d.* | |
|  | 1r | *Lactobacillus plantarum* | [1.871](file:///D:\Diplome\Jo%C5%BEi%20Omahen-probioti%C4%8Dni%20izdelki%20Lek\Maldi%20rezultati\Bruker%20Daltonik%20MALDI%20Biotyper%20Classification%20Results.htm#ID0EA) | *L. plantarum* | *n.d.* | |
|  | 1s | ***no peaks found*** | [< 0](file:///D:\Diplome\Jo%C5%BEi%20Omahen-probioti%C4%8Dni%20izdelki%20Lek\Maldi%20rezultati\Bruker%20Daltonik%20MALDI%20Biotyper%20Classification%20Results.htm#ID0EA) | *L. acidophilus* | *n.d.* | |
|  | 1t | [*Lactobacillus acidophilus*](file:///D:\Diplome\Jo%C5%BEi%20Omahen-probioti%C4%8Dni%20izdelki%20Lek\Maldi%20rezultati\Bruker%20Daltonik%20MALDI%20Biotyper%20Classification%20Results.htm#ID0EOCA) | [2.081](file:///D:\Diplome\Jo%C5%BEi%20Omahen-probioti%C4%8Dni%20izdelki%20Lek\Maldi%20rezultati\Bruker%20Daltonik%20MALDI%20Biotyper%20Classification%20Results.htm#ID0EA) | *L. acidophilus* | *n.d.* | |
|  | 1u | *Lactobacillus plantarum* | [2.083](file:///D:\Diplome\Jo%C5%BEi%20Omahen-probioti%C4%8Dni%20izdelki%20Lek\Maldi%20rezultati\Bruker%20Daltonik%20MALDI%20Biotyper%20Classification%20Results.htm#ID0EA) | *L. plantarum* | *n.d.* | |
|  | 1v | *Lactobacillus zeae or*  *L. casei* | [2.168](file:///D:\Diplome\Jo%C5%BEi%20Omahen-probioti%C4%8Dni%20izdelki%20Lek\Maldi%20rezultati\Bruker%20Daltonik%20MALDI%20Biotyper%20Classification%20Results.htm#ID0EA)  2.15 | *L. casei* | *L. casei* | |
|  | 1a2 | *Bifidobacterium animalis* | [2.175](file:///D:\Diplome\Jo%C5%BEi%20Omahen-probioti%C4%8Dni%20izdelki%20Lek\Maldi%20rezultati\Bruker%20Daltonik%20MALDI%20Biotyper%20Classification%20Results.htm#ID0EA) | *B. lactis* | *n.d.* | |
|  | 1b2 | *Bifidobacterium animalis* | [2.25](file:///D:\Diplome\Jo%C5%BEi%20Omahen-probioti%C4%8Dni%20izdelki%20Lek\Maldi%20rezultati\Bruker%20Daltonik%20MALDI%20Biotyper%20Classification%20Results.htm#ID0EA) | *B. lactis* | *n.d.* | |
|  | 1c2 | *Bifidobacterium animalis* | [2.29](file:///D:\Diplome\Jo%C5%BEi%20Omahen-probioti%C4%8Dni%20izdelki%20Lek\Maldi%20rezultati\Bruker%20Daltonik%20MALDI%20Biotyper%20Classification%20Results.htm#ID0EA) | *B. lactis* | *n.d.* | |
|  | 1d2 | *Bifidobacterium animalis* | [2.216](file:///D:\Diplome\Jo%C5%BEi%20Omahen-probioti%C4%8Dni%20izdelki%20Lek\Maldi%20rezultati\Bruker%20Daltonik%20MALDI%20Biotyper%20Classification%20Results.htm#ID0EA) | *B. lactis* | *n.d.* | |
|  | 1e2 | *Bifidobacterium animalis* | [2.229](file:///D:\Diplome\Jo%C5%BEi%20Omahen-probioti%C4%8Dni%20izdelki%20Lek\Maldi%20rezultati\Bruker%20Daltonik%20MALDI%20Biotyper%20Classification%20Results.htm#ID0EA) | *B. lactis* | *n.d.* | |
|  | 1f2 | *Bifidobacterium animalis* | [2.183](file:///D:\Diplome\Jo%C5%BEi%20Omahen-probioti%C4%8Dni%20izdelki%20Lek\Maldi%20rezultati\Bruker%20Daltonik%20MALDI%20Biotyper%20Classification%20Results.htm#ID0EA) | *B. lactis* | *n.d.* | |
|  | 1g2 | *Bifidobacterium animalis* | [2.151](file:///D:\Diplome\Jo%C5%BEi%20Omahen-probioti%C4%8Dni%20izdelki%20Lek\Maldi%20rezultati\Bruker%20Daltonik%20MALDI%20Biotyper%20Classification%20Results.htm#ID0EA) | *B. lactis* | *n.d.* | |
|  | 1h2 | *Bifidobacterium animalis* | [2.144](file:///D:\Diplome\Jo%C5%BEi%20Omahen-probioti%C4%8Dni%20izdelki%20Lek\Maldi%20rezultati\Bruker%20Daltonik%20MALDI%20Biotyper%20Classification%20Results.htm#ID0EA) | *B. lactis* | *n.d.* | |
|  | 1i2 | *Bifidobacterium animalis* | [2.227](file:///D:\Diplome\Jo%C5%BEi%20Omahen-probioti%C4%8Dni%20izdelki%20Lek\Maldi%20rezultati\Bruker%20Daltonik%20MALDI%20Biotyper%20Classification%20Results.htm#ID0EA) | *B. lactis* | *n.d.* | |
| 2 | 2a | *Bifidobacterium animalis* | [2.259](file:///D:\Diplome\Jo%C5%BEi%20Omahen-probioti%C4%8Dni%20izdelki%20Lek\Maldi%20rezultati\Bruker%20Daltonik%20MALDI%20Biotyper%20Classification%20Results.htm#ID0EA) | *B. lactis* | *n.d.* | |
|  | 2b | *Bifidobacterium animalis* | [2.343](file:///D:\Diplome\Jo%C5%BEi%20Omahen-probioti%C4%8Dni%20izdelki%20Lek\Maldi%20rezultati\Bruker%20Daltonik%20MALDI%20Biotyper%20Classification%20Results.htm#ID0EA) | *B. lactis* | *n.d.* | |
|  | 2c | *Bifidobacterium animalis* | [2.215](file:///D:\Diplome\Jo%C5%BEi%20Omahen-probioti%C4%8Dni%20izdelki%20Lek\Maldi%20rezultati\Bruker%20Daltonik%20MALDI%20Biotyper%20Classification%20Results.htm#ID0EA) | *B. lactis* | *n.d.* | |
|  | 2d | *Bifidobacterium animalis* | [2.157](file:///D:\Diplome\Jo%C5%BEi%20Omahen-probioti%C4%8Dni%20izdelki%20Lek\Maldi%20rezultati\Bruker%20Daltonik%20MALDI%20Biotyper%20Classification%20Results.htm#ID0EA) | *B. lactis* | *n.d.* | |
|  | 2e | *Bifidobacterium animalis* | [2.189](file:///D:\Diplome\Jo%C5%BEi%20Omahen-probioti%C4%8Dni%20izdelki%20Lek\Maldi%20rezultati\Bruker%20Daltonik%20MALDI%20Biotyper%20Classification%20Results.htm#ID0EA) | *B. lactis* | *n.d.* | |
|  | 2f | *Bifidobacterium animalis* | [2.216](file:///D:\Diplome\Jo%C5%BEi%20Omahen-probioti%C4%8Dni%20izdelki%20Lek\Maldi%20rezultati\Bruker%20Daltonik%20MALDI%20Biotyper%20Classification%20Results.htm#ID0EA) | *B. lactis* | *n.d.* | |
|  | 2g | *Lactobacillus plantarum* | [1.937](file:///D:\Diplome\Jo%C5%BEi%20Omahen-probioti%C4%8Dni%20izdelki%20Lek\Maldi%20rezultati\Bruker%20Daltonik%20MALDI%20Biotyper%20Classification%20Results.htm#ID0EA) | *L. plantarum* | *n.d.* | |
|  | 2h | *Lactobacillus paracasei* | [2.28](file:///D:\Diplome\Jo%C5%BEi%20Omahen-probioti%C4%8Dni%20izdelki%20Lek\Maldi%20rezultati\Bruker%20Daltonik%20MALDI%20Biotyper%20Classification%20Results.htm#ID0EA) | *L. paracasei* | *n.d.* | |
|  | 2i | [*Lactobacillus acidophilus*](file:///D:\Diplome\Jo%C5%BEi%20Omahen-probioti%C4%8Dni%20izdelki%20Lek\Maldi%20rezultati\Bruker%20Daltonik%20MALDI%20Biotyper%20Classification%20Results.htm#ID0EOCA) | [2.371](file:///D:\Diplome\Jo%C5%BEi%20Omahen-probioti%C4%8Dni%20izdelki%20Lek\Maldi%20rezultati\Bruker%20Daltonik%20MALDI%20Biotyper%20Classification%20Results.htm#ID0EA) | *L. acidophilus* | *n.d.* | |
|  | 2j | [*Lactobacillus acidophilus*](file:///D:\Diplome\Jo%C5%BEi%20Omahen-probioti%C4%8Dni%20izdelki%20Lek\Maldi%20rezultati\Bruker%20Daltonik%20MALDI%20Biotyper%20Classification%20Results.htm#ID0EOCA) | [2.361](file:///D:\Diplome\Jo%C5%BEi%20Omahen-probioti%C4%8Dni%20izdelki%20Lek\Maldi%20rezultati\Bruker%20Daltonik%20MALDI%20Biotyper%20Classification%20Results.htm#ID0EA) | *L. acidophilus* | *n.d.* | |
|  | 2k | *Lactobacillus plantarum* | [2.161](file:///D:\Diplome\Jo%C5%BEi%20Omahen-probioti%C4%8Dni%20izdelki%20Lek\Maldi%20rezultati\Bruker%20Daltonik%20MALDI%20Biotyper%20Classification%20Results.htm#ID0EA) | *L. plantarum* | *n.d.* | |
|  | 2l | *Lactobacillus plantarum* | [2.047](file:///D:\Diplome\Jo%C5%BEi%20Omahen-probioti%C4%8Dni%20izdelki%20Lek\Maldi%20rezultati\Bruker%20Daltonik%20MALDI%20Biotyper%20Classification%20Results.htm#ID0EA) | *L. plantarum* | *n.d.* | |
|  | 2m | *Lactobacillus salivarius* | [2.417](file:///D:\Diplome\Jo%C5%BEi%20Omahen-probioti%C4%8Dni%20izdelki%20Lek\Maldi%20rezultati\Bruker%20Daltonik%20MALDI%20Biotyper%20Classification%20Results.htm#ID0EA) | *L. salivarius* | *n.d.* | |
|  | 2n | *Lactobacillus paracasei* | [2.355](file:///D:\Diplome\Jo%C5%BEi%20Omahen-probioti%C4%8Dni%20izdelki%20Lek\Maldi%20rezultati\Bruker%20Daltonik%20MALDI%20Biotyper%20Classification%20Results.htm#ID0EA) | *L. paracasei* | *n.d.* | |
|  | 2o | [*Lactobacillus acidophilus*](file:///D:\Diplome\Jo%C5%BEi%20Omahen-probioti%C4%8Dni%20izdelki%20Lek\Maldi%20rezultati\Bruker%20Daltonik%20MALDI%20Biotyper%20Classification%20Results.htm#ID0EOCA) | [2.37](file:///D:\Diplome\Jo%C5%BEi%20Omahen-probioti%C4%8Dni%20izdelki%20Lek\Maldi%20rezultati\Bruker%20Daltonik%20MALDI%20Biotyper%20Classification%20Results.htm#ID0EA) | *L. acidophilus* | *n.d.* | |
|  | 2p | [*Lactobacillus acidophilus*](file:///D:\Diplome\Jo%C5%BEi%20Omahen-probioti%C4%8Dni%20izdelki%20Lek\Maldi%20rezultati\Bruker%20Daltonik%20MALDI%20Biotyper%20Classification%20Results.htm#ID0EOCA) | [2.412](file:///D:\Diplome\Jo%C5%BEi%20Omahen-probioti%C4%8Dni%20izdelki%20Lek\Maldi%20rezultati\Bruker%20Daltonik%20MALDI%20Biotyper%20Classification%20Results.htm#ID0EA) | *L. acidophilus* | *n.d.* | |
|  | 2r | [*Lactobacillus acidophilus*](file:///D:\Diplome\Jo%C5%BEi%20Omahen-probioti%C4%8Dni%20izdelki%20Lek\Maldi%20rezultati\Bruker%20Daltonik%20MALDI%20Biotyper%20Classification%20Results.htm#ID0EOCA) | [2.359](file:///D:\Diplome\Jo%C5%BEi%20Omahen-probioti%C4%8Dni%20izdelki%20Lek\Maldi%20rezultati\Bruker%20Daltonik%20MALDI%20Biotyper%20Classification%20Results.htm#ID0EA) | *L. acidophilus* | *n.d.* | |
|  | 2s | *Lactobacillus plantarum* | [2.167](file:///D:\Diplome\Jo%C5%BEi%20Omahen-probioti%C4%8Dni%20izdelki%20Lek\Maldi%20rezultati\Bruker%20Daltonik%20MALDI%20Biotyper%20Classification%20Results.htm#ID0EA) | *L. plantarum* | *n.d.* | |
|  | 2t | *Lactobacillus plantarum* | [1.93](file:///D:\Diplome\Jo%C5%BEi%20Omahen-probioti%C4%8Dni%20izdelki%20Lek\Maldi%20rezultati\Bruker%20Daltonik%20MALDI%20Biotyper%20Classification%20Results.htm#ID0EA) | *L. plantarum* | *n.d.* | |
|  | 2u | [*Lactobacillus acidophilus*](file:///D:\Diplome\Jo%C5%BEi%20Omahen-probioti%C4%8Dni%20izdelki%20Lek\Maldi%20rezultati\Bruker%20Daltonik%20MALDI%20Biotyper%20Classification%20Results.htm#ID0EOCA) | [2.205](file:///D:\Diplome\Jo%C5%BEi%20Omahen-probioti%C4%8Dni%20izdelki%20Lek\Maldi%20rezultati\Bruker%20Daltonik%20MALDI%20Biotyper%20Classification%20Results.htm#ID0EA) | *L. acidophilus* | *n.d.* | |
|  | 2v | [*Lactobacillus acidophilus*](file:///D:\Diplome\Jo%C5%BEi%20Omahen-probioti%C4%8Dni%20izdelki%20Lek\Maldi%20rezultati\Bruker%20Daltonik%20MALDI%20Biotyper%20Classification%20Results.htm#ID0EOCA) | [2.405](file:///D:\Diplome\Jo%C5%BEi%20Omahen-probioti%C4%8Dni%20izdelki%20Lek\Maldi%20rezultati\Bruker%20Daltonik%20MALDI%20Biotyper%20Classification%20Results.htm#ID0EA) | *n.d.* | [*Lactobacillus acidophilus*](file:///D:\Diplome\Jo%C5%BEi%20Omahen-probioti%C4%8Dni%20izdelki%20Lek\Maldi%20rezultati\Bruker%20Daltonik%20MALDI%20Biotyper%20Classification%20Results.htm#ID0EOCA) | |
|  | 2z | *Lactococcus lactis* | [2.214](file:///D:\Diplome\Jo%C5%BEi%20Omahen-probioti%C4%8Dni%20izdelki%20Lek\Maldi%20rezultati\Bruker%20Daltonik%20MALDI%20Biotyper%20Classification%20Results.htm#ID0EA) | *Lc. lactis* | *n.d.* | |
|  | 2x | *Lactococcus lactis* | [2.211](file:///D:\Diplome\Jo%C5%BEi%20Omahen-probioti%C4%8Dni%20izdelki%20Lek\Maldi%20rezultati\Bruker%20Daltonik%20MALDI%20Biotyper%20Classification%20Results.htm#ID0EA) | *Lc. lactis* | *n.d.* | |
|  | 2y | *Lactococcus lactis* | [2.288](file:///D:\Diplome\Jo%C5%BEi%20Omahen-probioti%C4%8Dni%20izdelki%20Lek\Maldi%20rezultati\Bruker%20Daltonik%20MALDI%20Biotyper%20Classification%20Results.htm#ID0EA) | *Lc. lactis* | *n.d.* | |
| 3 | 3a | *Lactobacillus paracasei* | [2.307](file:///D:\Diplome\Jo%C5%BEi%20Omahen-probioti%C4%8Dni%20izdelki%20Lek\Maldi%20rezultati\Bruker%20Daltonik%20MALDI%20Biotyper%20Classification%20Results.htm#ID0EA) | *L. paracasei* | *n.d.* | |
|  | 3b | *Lactobacillus paracasei* | [2.312](file:///D:\Diplome\Jo%C5%BEi%20Omahen-probioti%C4%8Dni%20izdelki%20Lek\Maldi%20rezultati\Bruker%20Daltonik%20MALDI%20Biotyper%20Classification%20Results.htm#ID0EA) | *L. paracasei* | *n.d.* | |
|  | 3c | *Lactobacillus paracasei* | [2.273](file:///D:\Diplome\Jo%C5%BEi%20Omahen-probioti%C4%8Dni%20izdelki%20Lek\Maldi%20rezultati\Bruker%20Daltonik%20MALDI%20Biotyper%20Classification%20Results.htm#ID0EA) | *L. paracasei* | *n.d.* | |
|  | 3d | *Bifidobacterium longum* | [1.945](file:///D:\Diplome\Jo%C5%BEi%20Omahen-probioti%C4%8Dni%20izdelki%20Lek\Maldi%20rezultati\Bruker%20Daltonik%20MALDI%20Biotyper%20Classification%20Results.htm#ID0EA) | *B. longum* | *n.d.* | |
|  | 3e | *Bifidobacterium longum* | [1.984](file:///D:\Diplome\Jo%C5%BEi%20Omahen-probioti%C4%8Dni%20izdelki%20Lek\Maldi%20rezultati\Bruker%20Daltonik%20MALDI%20Biotyper%20Classification%20Results.htm#ID0EA) | *B. longum* | *n.d.* | |
|  | 3f | *Bifidobacterium longum* | [2.075](file:///D:\Diplome\Jo%C5%BEi%20Omahen-probioti%C4%8Dni%20izdelki%20Lek\Maldi%20rezultati\Bruker%20Daltonik%20MALDI%20Biotyper%20Classification%20Results.htm#ID0EA) | *B. longum* | *n.d.* | |
|  | 3g | *Lactobacillus rhamnosus* | [1.899](file:///D:\Diplome\Jo%C5%BEi%20Omahen-probioti%C4%8Dni%20izdelki%20Lek\Maldi%20rezultati\Bruker%20Daltonik%20MALDI%20Biotyper%20Classification%20Results.htm#ID0EA) | *L. rhamnosus* | *n.d.* | |
|  | 3h | *Lactobacillus zeae* | [1.965](file:///D:\Diplome\Jo%C5%BEi%20Omahen-probioti%C4%8Dni%20izdelki%20Lek\Maldi%20rezultati\Bruker%20Daltonik%20MALDI%20Biotyper%20Classification%20Results.htm#ID0EA) | *L. rhamnosus* | *L. rhamnosus* | |
|  | 3i | [*Lactobacillus acidophilus*](file:///D:\Diplome\Jo%C5%BEi%20Omahen-probioti%C4%8Dni%20izdelki%20Lek\Maldi%20rezultati\Bruker%20Daltonik%20MALDI%20Biotyper%20Classification%20Results.htm#ID0EOCA) | [2.321](file:///D:\Diplome\Jo%C5%BEi%20Omahen-probioti%C4%8Dni%20izdelki%20Lek\Maldi%20rezultati\Bruker%20Daltonik%20MALDI%20Biotyper%20Classification%20Results.htm#ID0EA) | *L. acidophilus* | *n.d.* | |
|  | 3j | [*Lactobacillus acidophilus*](file:///D:\Diplome\Jo%C5%BEi%20Omahen-probioti%C4%8Dni%20izdelki%20Lek\Maldi%20rezultati\Bruker%20Daltonik%20MALDI%20Biotyper%20Classification%20Results.htm#ID0EOCA) | [2.215](file:///D:\Diplome\Jo%C5%BEi%20Omahen-probioti%C4%8Dni%20izdelki%20Lek\Maldi%20rezultati\Bruker%20Daltonik%20MALDI%20Biotyper%20Classification%20Results.htm#ID0EA) | *L. acidophilus* | *n.d.* | |
|  | 3k | *Lactobacillus casei* | [1.847](file:///D:\Diplome\Jo%C5%BEi%20Omahen-probioti%C4%8Dni%20izdelki%20Lek\Maldi%20rezultati\Bruker%20Daltonik%20MALDI%20Biotyper%20Classification%20Results.htm#ID0EA) | *L. rhamnosus* | *L. rhamnosus* | |
|  | 3l | [*Lactobacillus acidophilus*](file:///D:\Diplome\Jo%C5%BEi%20Omahen-probioti%C4%8Dni%20izdelki%20Lek\Maldi%20rezultati\Bruker%20Daltonik%20MALDI%20Biotyper%20Classification%20Results.htm#ID0EOCA) | [1.903](file:///D:\Diplome\Jo%C5%BEi%20Omahen-probioti%C4%8Dni%20izdelki%20Lek\Maldi%20rezultati\Bruker%20Daltonik%20MALDI%20Biotyper%20Classification%20Results.htm#ID0EA) | *L. acidophilus* | *n.d.* | |
|  | 3m | [*Lactobacillus acidophilus*](file:///D:\Diplome\Jo%C5%BEi%20Omahen-probioti%C4%8Dni%20izdelki%20Lek\Maldi%20rezultati\Bruker%20Daltonik%20MALDI%20Biotyper%20Classification%20Results.htm#ID0EYCA) | [2.409](file:///D:\Diplome\Jo%C5%BEi%20Omahen-probioti%C4%8Dni%20izdelki%20Lek\Maldi%20rezultati\Bruker%20Daltonik%20MALDI%20Biotyper%20Classification%20Results.htm#ID0EA) | *L. acidophilus* | *n.d.* | |
|  | 3n | *Lactobacillus zeae* | [1.743](file:///D:\Diplome\Jo%C5%BEi%20Omahen-probioti%C4%8Dni%20izdelki%20Lek\Maldi%20rezultati\Bruker%20Daltonik%20MALDI%20Biotyper%20Classification%20Results.htm#ID0EA) | *L. rhamnosus* | *L. rhamnosus* | |
|  | 3o | *Lactobacillus rhamnosus* | [2.22](file:///D:\Diplome\Jo%C5%BEi%20Omahen-probioti%C4%8Dni%20izdelki%20Lek\Maldi%20rezultati\Bruker%20Daltonik%20MALDI%20Biotyper%20Classification%20Results.htm#ID0EA) | *L. rhamnosus* | *n.d.* | |
|  | 3p | [*Lactobacillus acidophilus*](file:///D:\Diplome\Jo%C5%BEi%20Omahen-probioti%C4%8Dni%20izdelki%20Lek\Maldi%20rezultati\Bruker%20Daltonik%20MALDI%20Biotyper%20Classification%20Results.htm#ID0EOCA) | [2.461](file:///D:\Diplome\Jo%C5%BEi%20Omahen-probioti%C4%8Dni%20izdelki%20Lek\Maldi%20rezultati\Bruker%20Daltonik%20MALDI%20Biotyper%20Classification%20Results.htm#ID0EA) | *L. acidophilus* | *n.d.* | |
|  | 3r | [*Lactobacillus acidophilus*](file:///D:\Diplome\Jo%C5%BEi%20Omahen-probioti%C4%8Dni%20izdelki%20Lek\Maldi%20rezultati\Bruker%20Daltonik%20MALDI%20Biotyper%20Classification%20Results.htm#ID0EYCA) | [2.494](file:///D:\Diplome\Jo%C5%BEi%20Omahen-probioti%C4%8Dni%20izdelki%20Lek\Maldi%20rezultati\Bruker%20Daltonik%20MALDI%20Biotyper%20Classification%20Results.htm#ID0EA) | *L. acidophilus* | *n.d.* | |
|  | 3s | *Lactobacillus casei* | [2.042](file:///D:\Diplome\Jo%C5%BEi%20Omahen-probioti%C4%8Dni%20izdelki%20Lek\Maldi%20rezultati\Bruker%20Daltonik%20MALDI%20Biotyper%20Classification%20Results.htm#ID0EA) | *L. rhamnosus* | *L. rhamnosus* | |
|  | 3t | [*Lactobacillus acidophilus*](file:///D:\Diplome\Jo%C5%BEi%20Omahen-probioti%C4%8Dni%20izdelki%20Lek\Maldi%20rezultati\Bruker%20Daltonik%20MALDI%20Biotyper%20Classification%20Results.htm#ID0EYCA) | [2.391](file:///D:\Diplome\Jo%C5%BEi%20Omahen-probioti%C4%8Dni%20izdelki%20Lek\Maldi%20rezultati\Bruker%20Daltonik%20MALDI%20Biotyper%20Classification%20Results.htm#ID0EA) | *L. acidophilus* | *n.d.* | |
|  | 3u | *Lactobacillus rhamnosus* | [1.856](file:///D:\Diplome\Jo%C5%BEi%20Omahen-probioti%C4%8Dni%20izdelki%20Lek\Maldi%20rezultati\Bruker%20Daltonik%20MALDI%20Biotyper%20Classification%20Results.htm#ID0EA) | *L. rhamnosus* | *n.d.* | |
|  | 3v | *Lactobacillus zeae* | [1.885](file:///D:\Diplome\Jo%C5%BEi%20Omahen-probioti%C4%8Dni%20izdelki%20Lek\Maldi%20rezultati\Bruker%20Daltonik%20MALDI%20Biotyper%20Classification%20Results.htm#ID0EA) | *L. rhamnosus* | *L. rhamnosus* | |
| 4 | 4a | *Lactobacillus gasseri* | [2.458](file:///D:\Diplome\Jo%C5%BEi%20Omahen-probioti%C4%8Dni%20izdelki%20Lek\Maldi%20rezultati\Bruker%20Daltonik%20MALDI%20Biotyper%20Classification%20Results.htm#ID0EA) | *L. gasseri* | *n.d.* | |
|  | 4b | [*Lactobacillus acidophilus*](file:///D:\Diplome\Jo%C5%BEi%20Omahen-probioti%C4%8Dni%20izdelki%20Lek\Maldi%20rezultati\Bruker%20Daltonik%20MALDI%20Biotyper%20Classification%20Results.htm#ID0EOCA) | [2.472](file:///D:\Diplome\Jo%C5%BEi%20Omahen-probioti%C4%8Dni%20izdelki%20Lek\Maldi%20rezultati\Bruker%20Daltonik%20MALDI%20Biotyper%20Classification%20Results.htm#ID0EA) | *L. acidophilus* | *n.d.* | |
|  | 4c | *Lactobacillus zeae* | [1.867](file:///D:\Diplome\Jo%C5%BEi%20Omahen-probioti%C4%8Dni%20izdelki%20Lek\Maldi%20rezultati\Bruker%20Daltonik%20MALDI%20Biotyper%20Classification%20Results.htm#ID0EA) | *L. rhamnosus* | *L. rhamnosus* | |
|  | 4d | *Lactobacillus paracasei* | [2.275](file:///D:\Diplome\Jo%C5%BEi%20Omahen-probioti%C4%8Dni%20izdelki%20Lek\Maldi%20rezultati\Bruker%20Daltonik%20MALDI%20Biotyper%20Classification%20Results.htm#ID0EA) | *L. paracasei* | *n.d.* | |
|  | 4e | *Lactobacillus zeae* | [1.765](file:///D:\Diplome\Jo%C5%BEi%20Omahen-probioti%C4%8Dni%20izdelki%20Lek\Maldi%20rezultati\Bruker%20Daltonik%20MALDI%20Biotyper%20Classification%20Results.htm#ID0EA) | *L. rhamnosus* | *L. rhamnosus* | |
|  | 4f | [*Lactobacillus acidophilus*](file:///D:\Diplome\Jo%C5%BEi%20Omahen-probioti%C4%8Dni%20izdelki%20Lek\Maldi%20rezultati\Bruker%20Daltonik%20MALDI%20Biotyper%20Classification%20Results.htm#ID0EOCA) | [2.404](file:///D:\Diplome\Jo%C5%BEi%20Omahen-probioti%C4%8Dni%20izdelki%20Lek\Maldi%20rezultati\Bruker%20Daltonik%20MALDI%20Biotyper%20Classification%20Results.htm#ID0EA) | *L. acidophilus* | *n.d.* | |
|  | 4g | *Lactobacillus paracasei* | [1.822](file:///D:\Diplome\Jo%C5%BEi%20Omahen-probioti%C4%8Dni%20izdelki%20Lek\Maldi%20rezultati\Bruker%20Daltonik%20MALDI%20Biotyper%20Classification%20Results.htm#ID0EA) | *L. paracasei* | *n.d.* | |
|  | 4h | *Lactobacillus gasseri* | [2.332](file:///D:\Diplome\Jo%C5%BEi%20Omahen-probioti%C4%8Dni%20izdelki%20Lek\Maldi%20rezultati\Bruker%20Daltonik%20MALDI%20Biotyper%20Classification%20Results.htm#ID0EA) | *L. gasseri* | *n.d.* | |
|  | 4i | *Lactobacillus paracasei* | [2.323](file:///D:\Diplome\Jo%C5%BEi%20Omahen-probioti%C4%8Dni%20izdelki%20Lek\Maldi%20rezultati\Bruker%20Daltonik%20MALDI%20Biotyper%20Classification%20Results.htm#ID0EA) | *L. paracasei* | *n.d.* | |
|  | 4j | *Lactobacillus paracasei* | [2.343](file:///D:\Diplome\Jo%C5%BEi%20Omahen-probioti%C4%8Dni%20izdelki%20Lek\Maldi%20rezultati\Bruker%20Daltonik%20MALDI%20Biotyper%20Classification%20Results.htm#ID0EA) | *L. paracasei* | *n.d.* | |
|  | 4k | *Lactobacillus zeae* | [1.744](file:///D:\Diplome\Jo%C5%BEi%20Omahen-probioti%C4%8Dni%20izdelki%20Lek\Maldi%20rezultati\Bruker%20Daltonik%20MALDI%20Biotyper%20Classification%20Results.htm#ID0EA) | *L. rhamnosus* | *L. rhamnosus* | |
|  | 4l | *Lactobacillus zeae* | [1.729](file:///D:\Diplome\Jo%C5%BEi%20Omahen-probioti%C4%8Dni%20izdelki%20Lek\Maldi%20rezultati\Bruker%20Daltonik%20MALDI%20Biotyper%20Classification%20Results.htm#ID0EA) | *L. rhamnosus* | *L. rhamnosus* | |
|  | 4m | *Lactobacillus rhamnosus* | [1.876](file:///D:\Diplome\Jo%C5%BEi%20Omahen-probioti%C4%8Dni%20izdelki%20Lek\Maldi%20rezultati\Bruker%20Daltonik%20MALDI%20Biotyper%20Classification%20Results.htm#ID0EA) | *L. rhamnosus* | *n.d.* | |
|  | 4n | *Lactobacillus zeae* | [1.808](file:///D:\Diplome\Jo%C5%BEi%20Omahen-probioti%C4%8Dni%20izdelki%20Lek\Maldi%20rezultati\Bruker%20Daltonik%20MALDI%20Biotyper%20Classification%20Results.htm#ID0EA) | *L. rhamnosus* | *L. rhamnosus* | |
|  | 4o | *Lactobacillus paracasei* | [2.357](file:///D:\Diplome\Jo%C5%BEi%20Omahen-probioti%C4%8Dni%20izdelki%20Lek\Maldi%20rezultati\Bruker%20Daltonik%20MALDI%20Biotyper%20Classification%20Results.htm#ID0EA) | *L. paracasei* | *n.d.* | |
|  | 4p | *Lactobacillus paracasei* | [2.346](file:///D:\Diplome\Jo%C5%BEi%20Omahen-probioti%C4%8Dni%20izdelki%20Lek\Maldi%20rezultati\Bruker%20Daltonik%20MALDI%20Biotyper%20Classification%20Results.htm#ID0EA) | *L. paracasei* | *n.d.* | |
|  | 4r | *Lactobacillus zeae* | [1.774](file:///D:\Diplome\Jo%C5%BEi%20Omahen-probioti%C4%8Dni%20izdelki%20Lek\Maldi%20rezultati\Bruker%20Daltonik%20MALDI%20Biotyper%20Classification%20Results.htm#ID0EA) | *L. rhamnosus* | *L. rhamnosus* | |
|  | 4s | *Lactobacillus casei* | [2.203](file:///D:\Diplome\Jo%C5%BEi%20Omahen-probioti%C4%8Dni%20izdelki%20Lek\Maldi%20rezultati\Bruker%20Daltonik%20MALDI%20Biotyper%20Classification%20Results.htm#ID0EA) | *L. casei* | *n.d.* | |
|  | 4t | *Lactobacillus gasseri* | [2.478](file:///D:\Diplome\Jo%C5%BEi%20Omahen-probioti%C4%8Dni%20izdelki%20Lek\Maldi%20rezultati\Bruker%20Daltonik%20MALDI%20Biotyper%20Classification%20Results.htm#ID0EA) | *n.d.* | *n.d.* | |
|  | 4u | *Lactobacillus paracasei* | [2.267](file:///D:\Diplome\Jo%C5%BEi%20Omahen-probioti%C4%8Dni%20izdelki%20Lek\Maldi%20rezultati\Bruker%20Daltonik%20MALDI%20Biotyper%20Classification%20Results.htm#ID0EA) | *L. paracasei* | *n.d.* | |
|  | 4v | [*Lactobacillus acidophilus*](file:///D:\Diplome\Jo%C5%BEi%20Omahen-probioti%C4%8Dni%20izdelki%20Lek\Maldi%20rezultati\Bruker%20Daltonik%20MALDI%20Biotyper%20Classification%20Results.htm#ID0EOCA) | [2.368](file:///D:\Diplome\Jo%C5%BEi%20Omahen-probioti%C4%8Dni%20izdelki%20Lek\Maldi%20rezultati\Bruker%20Daltonik%20MALDI%20Biotyper%20Classification%20Results.htm#ID0EA) | *L. acidophilus* | *n.d.* | |
|  | 4a2 | *Lactobacillus paracasei* | [2.279](file:///D:\Diplome\Jo%C5%BEi%20Omahen-probioti%C4%8Dni%20izdelki%20Lek\Maldi%20rezultati\druga%20plosca%20Bruker%20Daltonik%20MALDI%20Biotyper%20Classification%20Results.htm#ID0EA) | *L. paracasei* | *n.d.* | |
|  | 4b2 | *Lactobacillus paracasei* | [2.334](file:///D:\Diplome\Jo%C5%BEi%20Omahen-probioti%C4%8Dni%20izdelki%20Lek\Maldi%20rezultati\druga%20plosca%20Bruker%20Daltonik%20MALDI%20Biotyper%20Classification%20Results.htm#ID0EA) | *L. paracasei* | *n.d.* | |
|  | 4c2 | *Lactobacillus paracasei* | [2.175](file:///D:\Diplome\Jo%C5%BEi%20Omahen-probioti%C4%8Dni%20izdelki%20Lek\Maldi%20rezultati\druga%20plosca%20Bruker%20Daltonik%20MALDI%20Biotyper%20Classification%20Results.htm#ID0EA) | *L. paracasei* | *n.d.* | |
|  | 4d2 | *Lactobacillus paracasei* | [2.287](file:///D:\Diplome\Jo%C5%BEi%20Omahen-probioti%C4%8Dni%20izdelki%20Lek\Maldi%20rezultati\druga%20plosca%20Bruker%20Daltonik%20MALDI%20Biotyper%20Classification%20Results.htm#ID0EA) | *L. paracasei* | *n.d.* | |
|  | 4e2 | *Lactobacillus paracasei* | [2.219](file:///D:\Diplome\Jo%C5%BEi%20Omahen-probioti%C4%8Dni%20izdelki%20Lek\Maldi%20rezultati\druga%20plosca%20Bruker%20Daltonik%20MALDI%20Biotyper%20Classification%20Results.htm#ID0EA) | *L. paracasei* | *n.d.* | |
|  | 4f2 | *Bifidobacterium animalis* | [2.109](file:///D:\Diplome\Jo%C5%BEi%20Omahen-probioti%C4%8Dni%20izdelki%20Lek\Maldi%20rezultati\druga%20plosca%20Bruker%20Daltonik%20MALDI%20Biotyper%20Classification%20Results.htm#ID0EA) | *B. lactis* | *n.d.* | |
|  | 4g2 | *Bifidobacterium animalis* | [2.069](file:///D:\Diplome\Jo%C5%BEi%20Omahen-probioti%C4%8Dni%20izdelki%20Lek\Maldi%20rezultati\druga%20plosca%20Bruker%20Daltonik%20MALDI%20Biotyper%20Classification%20Results.htm#ID0EA) | *B. lactis* | *n.d.* | |
|  | 4h2 | *Bifidobacterium animalis* | [1.723](file:///D:\Diplome\Jo%C5%BEi%20Omahen-probioti%C4%8Dni%20izdelki%20Lek\Maldi%20rezultati\druga%20plosca%20Bruker%20Daltonik%20MALDI%20Biotyper%20Classification%20Results.htm#ID0EA) | *B. lactis* | *n.d.* | |
|  | 4i2 | *Bifidobacterium bifidum* | [2.098](file:///D:\Diplome\Jo%C5%BEi%20Omahen-probioti%C4%8Dni%20izdelki%20Lek\Maldi%20rezultati\druga%20plosca%20Bruker%20Daltonik%20MALDI%20Biotyper%20Classification%20Results.htm#ID0EA) | *B. bifidum* | *n.d.* | |
|  | 4j2 | *Bifidobacterium animalis* | [1.833](file:///D:\Diplome\Jo%C5%BEi%20Omahen-probioti%C4%8Dni%20izdelki%20Lek\Maldi%20rezultati\druga%20plosca%20Bruker%20Daltonik%20MALDI%20Biotyper%20Classification%20Results.htm#ID0EA) | *B. lactis* | *n.d.* | |
|  | 4k2 | *Bifidobacterium breve* | [1.962](file:///D:\Diplome\Jo%C5%BEi%20Omahen-probioti%C4%8Dni%20izdelki%20Lek\Maldi%20rezultati\druga%20plosca%20Bruker%20Daltonik%20MALDI%20Biotyper%20Classification%20Results.htm#ID0EA) | *B. breve* | *n.d.* | |
|  | 4l2 | *Bifidobacterium animalis* | [2.14](file:///D:\Diplome\Jo%C5%BEi%20Omahen-probioti%C4%8Dni%20izdelki%20Lek\Maldi%20rezultati\druga%20plosca%20Bruker%20Daltonik%20MALDI%20Biotyper%20Classification%20Results.htm#ID0EA) | *B. lactis* | *n.d.* | |
|  | 4m2 | ***no peaks found*** | [< 0](file:///D:\Diplome\Jo%C5%BEi%20Omahen-probioti%C4%8Dni%20izdelki%20Lek\Maldi%20rezultati\druga%20plosca%20Bruker%20Daltonik%20MALDI%20Biotyper%20Classification%20Results.htm#ID0EA) | *B. lactis* | *n.d.* | |
|  | 4n2 | *Bifidobacterium animalis* | [2.004](file:///D:\Diplome\Jo%C5%BEi%20Omahen-probioti%C4%8Dni%20izdelki%20Lek\Maldi%20rezultati\druga%20plosca%20Bruker%20Daltonik%20MALDI%20Biotyper%20Classification%20Results.htm#ID0EA) | *B. lactis* | *n.d.* | |
|  | 4o2 | *Bifidobacterium breve* | [1.837](file:///D:\Diplome\Jo%C5%BEi%20Omahen-probioti%C4%8Dni%20izdelki%20Lek\Maldi%20rezultati\druga%20plosca%20Bruker%20Daltonik%20MALDI%20Biotyper%20Classification%20Results.htm#ID0EA) | *B. breve* | *n.d.* | |
|  | 4p2 | *Bifidobacterium animalis* | [2.015](file:///D:\Diplome\Jo%C5%BEi%20Omahen-probioti%C4%8Dni%20izdelki%20Lek\Maldi%20rezultati\druga%20plosca%20Bruker%20Daltonik%20MALDI%20Biotyper%20Classification%20Results.htm#ID0EA) | *B. lactis* | *n.d.* | |
|  | 4r2 | *Bifidobacterium animalis* | [2.221](file:///D:\Diplome\Jo%C5%BEi%20Omahen-probioti%C4%8Dni%20izdelki%20Lek\Maldi%20rezultati\druga%20plosca%20Bruker%20Daltonik%20MALDI%20Biotyper%20Classification%20Results.htm#ID0EA) | *B. lactis* | *n.d.* | |
|  | 4s2 | *Bifidobacterium animalis* | [1.903](file:///D:\Diplome\Jo%C5%BEi%20Omahen-probioti%C4%8Dni%20izdelki%20Lek\Maldi%20rezultati\druga%20plosca%20Bruker%20Daltonik%20MALDI%20Biotyper%20Classification%20Results.htm#ID0EA) | *B. lactis* | *n.d.* | |
|  | 4t2 | *Bifidobacterium animalis* | [2.001](file:///D:\Diplome\Jo%C5%BEi%20Omahen-probioti%C4%8Dni%20izdelki%20Lek\Maldi%20rezultati\druga%20plosca%20Bruker%20Daltonik%20MALDI%20Biotyper%20Classification%20Results.htm#ID0EA) | *B. lactis* | *n.d.* | |
|  | 4u2 | *Bifidobacterium animalis* | [2.455](file:///D:\Diplome\Jo%C5%BEi%20Omahen-probioti%C4%8Dni%20izdelki%20Lek\Maldi%20rezultati\druga%20plosca%20Bruker%20Daltonik%20MALDI%20Biotyper%20Classification%20Results.htm#ID0EA) | *B. lactis* | *n.d.* | |
|  | P4a | *Lactococcus lactis* | [2.151](file:///D:\Diplome\Jo%C5%BEi%20Omahen-probioti%C4%8Dni%20izdelki%20Lek\Maldi%20rezultati\BF%20Rodica_69%20izolatov_M17%20iz%20vzorca%204.htm#ID0EA) | *Lc. lactis* | *n.d.* | |
|  | P4b | *Lactococcus lactis* | [2.319](file:///D:\Diplome\Jo%C5%BEi%20Omahen-probioti%C4%8Dni%20izdelki%20Lek\Maldi%20rezultati\BF%20Rodica_69%20izolatov_M17%20iz%20vzorca%204.htm#ID0EA) | *Lc. lactis* | *n.d.* | |
|  | P4c | *Lactococcus lactis* | [2.206](file:///D:\Diplome\Jo%C5%BEi%20Omahen-probioti%C4%8Dni%20izdelki%20Lek\Maldi%20rezultati\BF%20Rodica_69%20izolatov_M17%20iz%20vzorca%204.htm#ID0EA) | *Lc. lactis* | *n.d.* | |
| 5 | 5a | [*Lactobacillus acidophilus*](file:///D:\Diplome\Jo%C5%BEi%20Omahen-probioti%C4%8Dni%20izdelki%20Lek\Maldi%20rezultati\druga%20plosca%20Bruker%20Daltonik%20MALDI%20Biotyper%20Classification%20Results.htm#ID0EGCA) | [2.453](file:///D:\Diplome\Jo%C5%BEi%20Omahen-probioti%C4%8Dni%20izdelki%20Lek\Maldi%20rezultati\druga%20plosca%20Bruker%20Daltonik%20MALDI%20Biotyper%20Classification%20Results.htm#ID0EA) | *L. acidophilus* | *n.d.* | |
|  | 5b | [*Lactobacillus acidophilus*](file:///D:\Diplome\Jo%C5%BEi%20Omahen-probioti%C4%8Dni%20izdelki%20Lek\Maldi%20rezultati\druga%20plosca%20Bruker%20Daltonik%20MALDI%20Biotyper%20Classification%20Results.htm#ID0EGCA) | [2.376](file:///D:\Diplome\Jo%C5%BEi%20Omahen-probioti%C4%8Dni%20izdelki%20Lek\Maldi%20rezultati\druga%20plosca%20Bruker%20Daltonik%20MALDI%20Biotyper%20Classification%20Results.htm#ID0EA) | *L. acidophilus* | *n.d.* | |
|  | 5c | [*Lactobacillus acidophilus*](file:///D:\Diplome\Jo%C5%BEi%20Omahen-probioti%C4%8Dni%20izdelki%20Lek\Maldi%20rezultati\druga%20plosca%20Bruker%20Daltonik%20MALDI%20Biotyper%20Classification%20Results.htm#ID0EGCA) | [2.227](file:///D:\Diplome\Jo%C5%BEi%20Omahen-probioti%C4%8Dni%20izdelki%20Lek\Maldi%20rezultati\druga%20plosca%20Bruker%20Daltonik%20MALDI%20Biotyper%20Classification%20Results.htm#ID0EA) | *L. acidophilus* | *n.d.* | |
|  | 5d | [*Lactobacillus acidophilus*](file:///D:\Diplome\Jo%C5%BEi%20Omahen-probioti%C4%8Dni%20izdelki%20Lek\Maldi%20rezultati\druga%20plosca%20Bruker%20Daltonik%20MALDI%20Biotyper%20Classification%20Results.htm#ID0EGCA) | [2.398](file:///D:\Diplome\Jo%C5%BEi%20Omahen-probioti%C4%8Dni%20izdelki%20Lek\Maldi%20rezultati\druga%20plosca%20Bruker%20Daltonik%20MALDI%20Biotyper%20Classification%20Results.htm#ID0EA) | *L. acidophilus* | *n.d.* | |
|  | 5e | [*Lactobacillus acidophilus*](file:///D:\Diplome\Jo%C5%BEi%20Omahen-probioti%C4%8Dni%20izdelki%20Lek\Maldi%20rezultati\druga%20plosca%20Bruker%20Daltonik%20MALDI%20Biotyper%20Classification%20Results.htm#ID0EGCA) | [2.391](file:///D:\Diplome\Jo%C5%BEi%20Omahen-probioti%C4%8Dni%20izdelki%20Lek\Maldi%20rezultati\druga%20plosca%20Bruker%20Daltonik%20MALDI%20Biotyper%20Classification%20Results.htm#ID0EA) | *L. acidophilus* | *n.d.* | |
|  | 5f | [*Lactobacillus acidophilus*](file:///D:\Diplome\Jo%C5%BEi%20Omahen-probioti%C4%8Dni%20izdelki%20Lek\Maldi%20rezultati\druga%20plosca%20Bruker%20Daltonik%20MALDI%20Biotyper%20Classification%20Results.htm#ID0EGCA) | [2.446](file:///D:\Diplome\Jo%C5%BEi%20Omahen-probioti%C4%8Dni%20izdelki%20Lek\Maldi%20rezultati\druga%20plosca%20Bruker%20Daltonik%20MALDI%20Biotyper%20Classification%20Results.htm#ID0EA) | *L. acidophilus* |  | |
|  | 5g | *Bifidobacterium animalis* | [2.053](file:///D:\Diplome\Jo%C5%BEi%20Omahen-probioti%C4%8Dni%20izdelki%20Lek\Maldi%20rezultati\druga%20plosca%20Bruker%20Daltonik%20MALDI%20Biotyper%20Classification%20Results.htm#ID0EA) | *B. lactis* | *n.d.* | |
|  | 5h | *Bifidobacterium animalis* | [2.292](file:///D:\Diplome\Jo%C5%BEi%20Omahen-probioti%C4%8Dni%20izdelki%20Lek\Maldi%20rezultati\druga%20plosca%20Bruker%20Daltonik%20MALDI%20Biotyper%20Classification%20Results.htm#ID0EA) | *B. lactis* | *n.d.* | |
|  | 5i | *Bifidobacterium animalis* | [2.134](file:///D:\Diplome\Jo%C5%BEi%20Omahen-probioti%C4%8Dni%20izdelki%20Lek\Maldi%20rezultati\druga%20plosca%20Bruker%20Daltonik%20MALDI%20Biotyper%20Classification%20Results.htm#ID0EA) | *B. lactis* | *n.d.* | |
|  | 5j | *Bifidobacterium animalis* | [2.335](file:///D:\Diplome\Jo%C5%BEi%20Omahen-probioti%C4%8Dni%20izdelki%20Lek\Maldi%20rezultati\druga%20plosca%20Bruker%20Daltonik%20MALDI%20Biotyper%20Classification%20Results.htm#ID0EA) | *B. lactis* | *n.d.* | |
|  | 5k | *Bifidobacterium animalis* | [2.164](file:///D:\Diplome\Jo%C5%BEi%20Omahen-probioti%C4%8Dni%20izdelki%20Lek\Maldi%20rezultati\druga%20plosca%20Bruker%20Daltonik%20MALDI%20Biotyper%20Classification%20Results.htm#ID0EA) | *B. lactis* | *n.d.* | |
|  | 5l | *Bifidobacterium animalis* | [2.191](file:///D:\Diplome\Jo%C5%BEi%20Omahen-probioti%C4%8Dni%20izdelki%20Lek\Maldi%20rezultati\druga%20plosca%20Bruker%20Daltonik%20MALDI%20Biotyper%20Classification%20Results.htm#ID0EA) | *B. lactis* | *n.d.* | |
| 6 | 6a | ***not reliable identification*** | [1.495](file:///D:\Diplome\Jo%C5%BEi%20Omahen-probioti%C4%8Dni%20izdelki%20Lek\Maldi%20rezultati\89%20izolatov%2020190426%20Bruker%20Daltonik%20MALDI%20Biotyper%20Classification%20Results.htm#ID0EA) | *Bac. coagulans* | *n.d.* | |
|  | 6b | ***not reliable identification*** | [1.225](file:///D:\Diplome\Jo%C5%BEi%20Omahen-probioti%C4%8Dni%20izdelki%20Lek\Maldi%20rezultati\89%20izolatov%2020190426%20Bruker%20Daltonik%20MALDI%20Biotyper%20Classification%20Results.htm#ID0EA) | *Bac. coagulans* | *n.d.* | |
|  | 6c | ***not reliable identification*** | [1.399](file:///D:\Diplome\Jo%C5%BEi%20Omahen-probioti%C4%8Dni%20izdelki%20Lek\Maldi%20rezultati\89%20izolatov%2020190426%20Bruker%20Daltonik%20MALDI%20Biotyper%20Classification%20Results.htm#ID0EA) | *Bac. coagulans* | *n.d.* | |
| 7 | 7a | *Streptococcus salivarius_ssp_thermophilus* | [1.725](file:///D:\Diplome\Jo%C5%BEi%20Omahen-probioti%C4%8Dni%20izdelki%20Lek\Maldi%20rezultati\Petra%20LEK%20Bruker%20Daltonik%20MALDI%20Biotyper%20Classification%20Results.htm#ID0EA) | *S. thermophilus* | *n.d.* | |
|  | 7b | *Streptococcus salivarius_ssp_thermophilus* | [1.931](file:///D:\Diplome\Jo%C5%BEi%20Omahen-probioti%C4%8Dni%20izdelki%20Lek\Maldi%20rezultati\Petra%20LEK%20Bruker%20Daltonik%20MALDI%20Biotyper%20Classification%20Results.htm#ID0EA) | *S. thermophilus* | *n.d.* | |
|  | 7c | *Streptococcus salivarius_ssp_thermophilus* | [2.009](file:///D:\Diplome\Jo%C5%BEi%20Omahen-probioti%C4%8Dni%20izdelki%20Lek\Maldi%20rezultati\Petra%20LEK%20Bruker%20Daltonik%20MALDI%20Biotyper%20Classification%20Results.htm#ID0EA) | *S. thermophilus* | *n.d.* | |
|  | 7d | *Bifidobacterium animalis* | [2.268](file:///D:\Diplome\Jo%C5%BEi%20Omahen-probioti%C4%8Dni%20izdelki%20Lek\Maldi%20rezultati\Petra%20LEK%20Bruker%20Daltonik%20MALDI%20Biotyper%20Classification%20Results.htm#ID0EA) | *B. lactis* | *n.d.* | |
|  | 7e | *Bifidobacterium animalis* | [2.324](file:///D:\Diplome\Jo%C5%BEi%20Omahen-probioti%C4%8Dni%20izdelki%20Lek\Maldi%20rezultati\Petra%20LEK%20Bruker%20Daltonik%20MALDI%20Biotyper%20Classification%20Results.htm#ID0EA) | *B. lactis* | *n.d.* | |
|  | 7f | *Bifidobacterium animalis* | [2.161](file:///D:\Diplome\Jo%C5%BEi%20Omahen-probioti%C4%8Dni%20izdelki%20Lek\Maldi%20rezultati\Petra%20LEK%20Bruker%20Daltonik%20MALDI%20Biotyper%20Classification%20Results.htm#ID0EA) | *B. lactis* | *n.d.* | |
|  | 7g | *Bifidobacterium animalis* | [2.178](file:///D:\Diplome\Jo%C5%BEi%20Omahen-probioti%C4%8Dni%20izdelki%20Lek\Maldi%20rezultati\Petra%20LEK%20Bruker%20Daltonik%20MALDI%20Biotyper%20Classification%20Results.htm#ID0EA) | *B. lactis* | *n.d.* | |
|  | 7h | *Bifidobacterium animalis* | [2.187](file:///D:\Diplome\Jo%C5%BEi%20Omahen-probioti%C4%8Dni%20izdelki%20Lek\Maldi%20rezultati\Petra%20LEK%20Bruker%20Daltonik%20MALDI%20Biotyper%20Classification%20Results.htm#ID0EA) | *B. lactis* | *n.d.* | |
|  | 7i | *Bifidobacterium animalis* | [1.835](file:///D:\Diplome\Jo%C5%BEi%20Omahen-probioti%C4%8Dni%20izdelki%20Lek\Maldi%20rezultati\Petra%20LEK%20Bruker%20Daltonik%20MALDI%20Biotyper%20Classification%20Results.htm#ID0EA) | *B. lactis* | *n.d.* | |
|  | 7j | [*Lactobacillus acidophilus*](file:///D:\Diplome\Jo%C5%BEi%20Omahen-probioti%C4%8Dni%20izdelki%20Lek\Maldi%20rezultati\Petra%20LEK%20Bruker%20Daltonik%20MALDI%20Biotyper%20Classification%20Results.htm#ID0E0BB0CA) | [1.997](file:///D:\Diplome\Jo%C5%BEi%20Omahen-probioti%C4%8Dni%20izdelki%20Lek\Maldi%20rezultati\Petra%20LEK%20Bruker%20Daltonik%20MALDI%20Biotyper%20Classification%20Results.htm#ID0EA) | *L. acidophilus* | *n.d.* | |
|  | 7k | *Lactobacillus rhamnosus* | [2.016](file:///D:\Diplome\Jo%C5%BEi%20Omahen-probioti%C4%8Dni%20izdelki%20Lek\Maldi%20rezultati\Petra%20LEK%20Bruker%20Daltonik%20MALDI%20Biotyper%20Classification%20Results.htm#ID0EA) | *L. rhamnosus* | *n.d.* | |
|  | 7l | [*Lactobacillus acidophilus*](file:///D:\Diplome\Jo%C5%BEi%20Omahen-probioti%C4%8Dni%20izdelki%20Lek\Maldi%20rezultati\Petra%20LEK%20Bruker%20Daltonik%20MALDI%20Biotyper%20Classification%20Results.htm#ID0EUCA) | [2.344](file:///D:\Diplome\Jo%C5%BEi%20Omahen-probioti%C4%8Dni%20izdelki%20Lek\Maldi%20rezultati\Petra%20LEK%20Bruker%20Daltonik%20MALDI%20Biotyper%20Classification%20Results.htm#ID0EA) | *n.d.* | *L. acidophilus* | |
|  | 7m | [*Lactobacillus acidophilus*](file:///D:\Diplome\Jo%C5%BEi%20Omahen-probioti%C4%8Dni%20izdelki%20Lek\Maldi%20rezultati\Petra%20LEK%20Bruker%20Daltonik%20MALDI%20Biotyper%20Classification%20Results.htm#ID0EUCA) | [2.324](file:///D:\Diplome\Jo%C5%BEi%20Omahen-probioti%C4%8Dni%20izdelki%20Lek\Maldi%20rezultati\Petra%20LEK%20Bruker%20Daltonik%20MALDI%20Biotyper%20Classification%20Results.htm#ID0EA) | *L. acidophilus* | *n.d.* | |
|  | 7n | *Lactobacillus zeae* | [1.716](file:///D:\Diplome\Jo%C5%BEi%20Omahen-probioti%C4%8Dni%20izdelki%20Lek\Maldi%20rezultati\Petra%20LEK%20Bruker%20Daltonik%20MALDI%20Biotyper%20Classification%20Results.htm#ID0EA) | *L. rhamnosus* | *L. rhamnosus* | |
|  | 7o | *Lactobacillus zeae* | [1.878](file:///D:\Diplome\Jo%C5%BEi%20Omahen-probioti%C4%8Dni%20izdelki%20Lek\Maldi%20rezultati\Petra%20LEK%20Bruker%20Daltonik%20MALDI%20Biotyper%20Classification%20Results.htm#ID0EA) | *L. rhamnosus* | *L. rhamnosus* | |
|  | 7p | [*Lactobacillus acidophilus*](file:///D:\Diplome\Jo%C5%BEi%20Omahen-probioti%C4%8Dni%20izdelki%20Lek\Maldi%20rezultati\Petra%20LEK%20Bruker%20Daltonik%20MALDI%20Biotyper%20Classification%20Results.htm#ID0EUCA) | [1.95](file:///D:\Diplome\Jo%C5%BEi%20Omahen-probioti%C4%8Dni%20izdelki%20Lek\Maldi%20rezultati\Petra%20LEK%20Bruker%20Daltonik%20MALDI%20Biotyper%20Classification%20Results.htm#ID0EA) | *L. acidophilus* | *n.d.* | |
|  | 7r | [*Lactobacillus acidophilus*](file:///D:\Diplome\Jo%C5%BEi%20Omahen-probioti%C4%8Dni%20izdelki%20Lek\Maldi%20rezultati\Petra%20LEK%20Bruker%20Daltonik%20MALDI%20Biotyper%20Classification%20Results.htm#ID0EUCA) | [2.216](file:///D:\Diplome\Jo%C5%BEi%20Omahen-probioti%C4%8Dni%20izdelki%20Lek\Maldi%20rezultati\Petra%20LEK%20Bruker%20Daltonik%20MALDI%20Biotyper%20Classification%20Results.htm#ID0EA) | *L. acidophilus* | *n.d.* | |
|  | 7s | [*Lactobacillus acidophilus*](file:///D:\Diplome\Jo%C5%BEi%20Omahen-probioti%C4%8Dni%20izdelki%20Lek\Maldi%20rezultati\Petra%20LEK%20Bruker%20Daltonik%20MALDI%20Biotyper%20Classification%20Results.htm#ID0E0BB0CA) | [2.344](file:///D:\Diplome\Jo%C5%BEi%20Omahen-probioti%C4%8Dni%20izdelki%20Lek\Maldi%20rezultati\Petra%20LEK%20Bruker%20Daltonik%20MALDI%20Biotyper%20Classification%20Results.htm#ID0EA) | *L. acidophilus* | *n.d.* | |
| 8 | 8a | *Streptococcus salivarius_ssp_thermophilus* | [1.896](file:///D:\Diplome\Jo%C5%BEi%20Omahen-probioti%C4%8Dni%20izdelki%20Lek\Maldi%20rezultati\89%20izolatov%2020190426%20Bruker%20Daltonik%20MALDI%20Biotyper%20Classification%20Results.htm#ID0EA) | *S. thermophilus* | *n.d.* | |
|  | 8b | *Streptococcus salivarius_ssp_thermophilus* | [1.997](file:///D:\Diplome\Jo%C5%BEi%20Omahen-probioti%C4%8Dni%20izdelki%20Lek\Maldi%20rezultati\89%20izolatov%2020190426%20Bruker%20Daltonik%20MALDI%20Biotyper%20Classification%20Results.htm#ID0EA) | *S. thermophilus* | *n.d.* | |
|  | 8c | *Bifidobacterium animalis* | [1.999](file:///D:\Diplome\Jo%C5%BEi%20Omahen-probioti%C4%8Dni%20izdelki%20Lek\Maldi%20rezultati\89%20izolatov%2020190426%20Bruker%20Daltonik%20MALDI%20Biotyper%20Classification%20Results.htm#ID0EA) | *n.d.* | *n.d.* | |
|  | 8d | ***no peaks found*** | [< 0](file:///D:\Diplome\Jo%C5%BEi%20Omahen-probioti%C4%8Dni%20izdelki%20Lek\Maldi%20rezultati\89%20izolatov%2020190426%20Bruker%20Daltonik%20MALDI%20Biotyper%20Classification%20Results.htm#ID0EA) | *B. lactis* | *n.d.* | |
|  | 8e | *Bifidobacterium animalis* | [2.152](file:///D:\Diplome\Jo%C5%BEi%20Omahen-probioti%C4%8Dni%20izdelki%20Lek\Maldi%20rezultati\89%20izolatov%2020190426%20Bruker%20Daltonik%20MALDI%20Biotyper%20Classification%20Results.htm#ID0EA) | *B. lactis* | *n.d.* | |
|  | 8f | *Bifidobacterium animalis* | [2.006](file:///D:\Diplome\Jo%C5%BEi%20Omahen-probioti%C4%8Dni%20izdelki%20Lek\Maldi%20rezultati\89%20izolatov%2020190426%20Bruker%20Daltonik%20MALDI%20Biotyper%20Classification%20Results.htm#ID0EA) | *B. lactis* | *n.d.* | |
|  | 8g | *Bifidobacterium animalis* | [1.956](file:///D:\Diplome\Jo%C5%BEi%20Omahen-probioti%C4%8Dni%20izdelki%20Lek\Maldi%20rezultati\89%20izolatov%2020190426%20Bruker%20Daltonik%20MALDI%20Biotyper%20Classification%20Results.htm#ID0EA) | *B. lactis* | *n.d.* | |
|  | 8h | *Bifidobacterium animalis* | [2.096](file:///D:\Diplome\Jo%C5%BEi%20Omahen-probioti%C4%8Dni%20izdelki%20Lek\Maldi%20rezultati\89%20izolatov%2020190426%20Bruker%20Daltonik%20MALDI%20Biotyper%20Classification%20Results.htm#ID0EA) | *B. lactis* | *n.d.* | |
|  | 8i | *Bifidobacterium breve* | [1.985](file:///D:\Diplome\Jo%C5%BEi%20Omahen-probioti%C4%8Dni%20izdelki%20Lek\Maldi%20rezultati\89%20izolatov%2020190426%20Bruker%20Daltonik%20MALDI%20Biotyper%20Classification%20Results.htm#ID0EA) | *B. breve* | *n.d.* | |
|  | 8j | *Lactobacillus reuteri* | [2.11](file:///D:\Diplome\Jo%C5%BEi%20Omahen-probioti%C4%8Dni%20izdelki%20Lek\Maldi%20rezultati\89%20izolatov%2020190426%20Bruker%20Daltonik%20MALDI%20Biotyper%20Classification%20Results.htm#ID0EA) | *n.d.* | *Lactobacillus reuteri* | |
|  | 8k | *Lactobacillus paracasei* | [2.371](file:///D:\Diplome\Jo%C5%BEi%20Omahen-probioti%C4%8Dni%20izdelki%20Lek\Maldi%20rezultati\89%20izolatov%2020190426%20Bruker%20Daltonik%20MALDI%20Biotyper%20Classification%20Results.htm#ID0EA) | *L. paracasei* | *n.d.* | |
|  | 8l | *Lactobacillus plantarum* | [1.879](file:///D:\Diplome\Jo%C5%BEi%20Omahen-probioti%C4%8Dni%20izdelki%20Lek\Maldi%20rezultati\89%20izolatov%2020190426%20Bruker%20Daltonik%20MALDI%20Biotyper%20Classification%20Results.htm#ID0EA) | *L. plantarum* | *n.d.* | |
|  | 8m | *Lactobacillus paracasei* | [2.343](file:///D:\Diplome\Jo%C5%BEi%20Omahen-probioti%C4%8Dni%20izdelki%20Lek\Maldi%20rezultati\89%20izolatov%2020190426%20Bruker%20Daltonik%20MALDI%20Biotyper%20Classification%20Results.htm#ID0EA) | *L. paracasei* | *n.d.* | |
|  | 8n | *Lactobacillus reuteri* | [2.114](file:///D:\Diplome\Jo%C5%BEi%20Omahen-probioti%C4%8Dni%20izdelki%20Lek\Maldi%20rezultati\89%20izolatov%2020190426%20Bruker%20Daltonik%20MALDI%20Biotyper%20Classification%20Results.htm#ID0EA) | *L. reuteri* | *n.d.* | |
|  | 8o | *Lactobacillus reuteri* | [1.979](file:///D:\Diplome\Jo%C5%BEi%20Omahen-probioti%C4%8Dni%20izdelki%20Lek\Maldi%20rezultati\89%20izolatov%2020190426%20Bruker%20Daltonik%20MALDI%20Biotyper%20Classification%20Results.htm#ID0EA) | *L. reuteri* | *n.d.* | |
|  | 8p | *Lactobacillus reuteri* | [1.967](file:///D:\Diplome\Jo%C5%BEi%20Omahen-probioti%C4%8Dni%20izdelki%20Lek\Maldi%20rezultati\89%20izolatov%2020190426%20Bruker%20Daltonik%20MALDI%20Biotyper%20Classification%20Results.htm#ID0EA) | *L. reuteri* | *n.d.* | |
|  | 8r | *Lactobacillus reuteri* | [2.139](file:///D:\Diplome\Jo%C5%BEi%20Omahen-probioti%C4%8Dni%20izdelki%20Lek\Maldi%20rezultati\89%20izolatov%2020190426%20Bruker%20Daltonik%20MALDI%20Biotyper%20Classification%20Results.htm#ID0EA) | *L. reuteri* | *n.d.* | |
|  | 8s | *Lactobacillus plantarum* | [1.911](file:///D:\Diplome\Jo%C5%BEi%20Omahen-probioti%C4%8Dni%20izdelki%20Lek\Maldi%20rezultati\89%20izolatov%2020190426%20Bruker%20Daltonik%20MALDI%20Biotyper%20Classification%20Results.htm#ID0EA) | *L. plantarum* | *n.d.* | |
|  | 8t | [*Lactobacillus acidophilus*](file:///D:\Diplome\Jo%C5%BEi%20Omahen-probioti%C4%8Dni%20izdelki%20Lek\Maldi%20rezultati\89%20izolatov%2020190426%20Bruker%20Daltonik%20MALDI%20Biotyper%20Classification%20Results.htm#ID0EWCA) | [2.184](file:///D:\Diplome\Jo%C5%BEi%20Omahen-probioti%C4%8Dni%20izdelki%20Lek\Maldi%20rezultati\89%20izolatov%2020190426%20Bruker%20Daltonik%20MALDI%20Biotyper%20Classification%20Results.htm#ID0EA) | *L. acidophilus* | *n.d.* | |
|  | 8u | [*Lactobacillus acidophilus*](file:///D:\Diplome\Jo%C5%BEi%20Omahen-probioti%C4%8Dni%20izdelki%20Lek\Maldi%20rezultati\89%20izolatov%2020190426%20Bruker%20Daltonik%20MALDI%20Biotyper%20Classification%20Results.htm#ID0E0HB0CA) | [2.177](file:///D:\Diplome\Jo%C5%BEi%20Omahen-probioti%C4%8Dni%20izdelki%20Lek\Maldi%20rezultati\89%20izolatov%2020190426%20Bruker%20Daltonik%20MALDI%20Biotyper%20Classification%20Results.htm#ID0EA) | *L. acidophilus* | *n.d.* | |
|  | 8v | [*Lactobacillus acidophilus*](file:///D:\Diplome\Jo%C5%BEi%20Omahen-probioti%C4%8Dni%20izdelki%20Lek\Maldi%20rezultati\89%20izolatov%2020190426%20Bruker%20Daltonik%20MALDI%20Biotyper%20Classification%20Results.htm#ID0EWCA) | [2.273](file:///D:\Diplome\Jo%C5%BEi%20Omahen-probioti%C4%8Dni%20izdelki%20Lek\Maldi%20rezultati\89%20izolatov%2020190426%20Bruker%20Daltonik%20MALDI%20Biotyper%20Classification%20Results.htm#ID0EA) | *L. acidophilus* | *n.d.* | |
|  | 8a2 | *Lactobacillus paracasei* | [2.401](file:///D:\Diplome\Jo%C5%BEi%20Omahen-probioti%C4%8Dni%20izdelki%20Lek\Maldi%20rezultati\89%20izolatov%2020190426%20Bruker%20Daltonik%20MALDI%20Biotyper%20Classification%20Results.htm#ID0EA) | *L. paracasei* | *n.d.* | |
|  | 8b2 | [*Lactobacillus acidophilus*](file:///D:\Diplome\Jo%C5%BEi%20Omahen-probioti%C4%8Dni%20izdelki%20Lek\Maldi%20rezultati\89%20izolatov%2020190426%20Bruker%20Daltonik%20MALDI%20Biotyper%20Classification%20Results.htm#ID0EWCA) | [2.214](file:///D:\Diplome\Jo%C5%BEi%20Omahen-probioti%C4%8Dni%20izdelki%20Lek\Maldi%20rezultati\89%20izolatov%2020190426%20Bruker%20Daltonik%20MALDI%20Biotyper%20Classification%20Results.htm#ID0EA) | *L. acidophilus* | *n.d.* | |
|  | 8c2 | *Lactobacillus paracasei* | [2.05](file:///D:\Diplome\Jo%C5%BEi%20Omahen-probioti%C4%8Dni%20izdelki%20Lek\Maldi%20rezultati\89%20izolatov%2020190426%20Bruker%20Daltonik%20MALDI%20Biotyper%20Classification%20Results.htm#ID0EA) | *L. paracasei* | *n.d.* | |
|  | 8d2 | *Lactobacillus paracasei* | [2.365](file:///D:\Diplome\Jo%C5%BEi%20Omahen-probioti%C4%8Dni%20izdelki%20Lek\Maldi%20rezultati\89%20izolatov%2020190426%20Bruker%20Daltonik%20MALDI%20Biotyper%20Classification%20Results.htm#ID0EA) | *L. paracasei* | *n.d.* | |
|  | 8e2 | *Lactobacillus paracasei* | [2.207](file:///D:\Diplome\Jo%C5%BEi%20Omahen-probioti%C4%8Dni%20izdelki%20Lek\Maldi%20rezultati\89%20izolatov%2020190426%20Bruker%20Daltonik%20MALDI%20Biotyper%20Classification%20Results.htm#ID0EA) | *L. paracasei* | *n.d.* | |
|  | 8g2 | *Lactobacillus plantarum* | [2.11](file:///D:\Diplome\Jo%C5%BEi%20Omahen-probioti%C4%8Dni%20izdelki%20Lek\Maldi%20rezultati\89%20izolatov%2020190426%20Bruker%20Daltonik%20MALDI%20Biotyper%20Classification%20Results.htm#ID0EA) | *L. plantarum* | *n.d.* | |
|  | 8f2 | *Lactobacillus paracasei* | [2.074](file:///D:\Diplome\Jo%C5%BEi%20Omahen-probioti%C4%8Dni%20izdelki%20Lek\Maldi%20rezultati\89%20izolatov%2020190426%20Bruker%20Daltonik%20MALDI%20Biotyper%20Classification%20Results.htm#ID0EA) | *L. paracasei* | *n.d.* | |
|  | 8h2 | [*Lactobacillus acidophilus*](file:///D:\Diplome\Jo%C5%BEi%20Omahen-probioti%C4%8Dni%20izdelki%20Lek\Maldi%20rezultati\89%20izolatov%2020190426%20Bruker%20Daltonik%20MALDI%20Biotyper%20Classification%20Results.htm#ID0EWCA) | [2.345](file:///D:\Diplome\Jo%C5%BEi%20Omahen-probioti%C4%8Dni%20izdelki%20Lek\Maldi%20rezultati\89%20izolatov%2020190426%20Bruker%20Daltonik%20MALDI%20Biotyper%20Classification%20Results.htm#ID0EA) | *L. acidophilus* | *n.d.* | |
|  | 8i2 | ***no peaks found*** | [< 0](file:///D:\Diplome\Jo%C5%BEi%20Omahen-probioti%C4%8Dni%20izdelki%20Lek\Maldi%20rezultati\89%20izolatov%2020190426%20Bruker%20Daltonik%20MALDI%20Biotyper%20Classification%20Results.htm#ID0EA) | *L. acidophilus* | *n.d.* | |
|  | 8j2 | *Lactobacillus plantarum* | [2.149](file:///D:\Diplome\Jo%C5%BEi%20Omahen-probioti%C4%8Dni%20izdelki%20Lek\Maldi%20rezultati\89%20izolatov%2020190426%20Bruker%20Daltonik%20MALDI%20Biotyper%20Classification%20Results.htm#ID0EA) | *L. plantarum* | *n.d.* | |
|  | 8k2 | *Lactobacillus plantarum* | [1.993](file:///D:\Diplome\Jo%C5%BEi%20Omahen-probioti%C4%8Dni%20izdelki%20Lek\Maldi%20rezultati\89%20izolatov%2020190426%20Bruker%20Daltonik%20MALDI%20Biotyper%20Classification%20Results.htm#ID0EA) | *L. plantarum* | *n.d.* | |
|  | 8l2 | *Lactobacillus plantarum* | [2.175](file:///D:\Diplome\Jo%C5%BEi%20Omahen-probioti%C4%8Dni%20izdelki%20Lek\Maldi%20rezultati\89%20izolatov%2020190426%20Bruker%20Daltonik%20MALDI%20Biotyper%20Classification%20Results.htm#ID0EA) | *L. plantarum* | *n.d.* | |
| 9 | 9a | *Lactobacillus rhamnosus* | [1.928](file:///D:\Diplome\Jo%C5%BEi%20Omahen-probioti%C4%8Dni%20izdelki%20Lek\Maldi%20rezultati\89%20izolatov%2020190426%20Bruker%20Daltonik%20MALDI%20Biotyper%20Classification%20Results.htm#ID0EA) | *L. rhamnosus* | *n.d.* | |
|  | 9b | *Lactobacillus rhamnosus* | [1.724](file:///D:\Diplome\Jo%C5%BEi%20Omahen-probioti%C4%8Dni%20izdelki%20Lek\Maldi%20rezultati\89%20izolatov%2020190426%20Bruker%20Daltonik%20MALDI%20Biotyper%20Classification%20Results.htm#ID0EA) | *L. rhamnosus* | *n.d.* | |
|  | 9c | *Lactobacillus rhamnosus* | [1.832](file:///D:\Diplome\Jo%C5%BEi%20Omahen-probioti%C4%8Dni%20izdelki%20Lek\Maldi%20rezultati\89%20izolatov%2020190426%20Bruker%20Daltonik%20MALDI%20Biotyper%20Classification%20Results.htm#ID0EA) | *L. rhamnosus* | *n.d.* | |
| 10 | 10a | *Lactobacillus reuteri* | [2.091](file:///D:\Diplome\Jo%C5%BEi%20Omahen-probioti%C4%8Dni%20izdelki%20Lek\Maldi%20rezultati\89%20izolatov%2020190426%20Bruker%20Daltonik%20MALDI%20Biotyper%20Classification%20Results.htm#ID0EA) | *L. reuteri* | *n.d.* | |
|  | 10b | *Lactobacillus reuteri* | [2.063](file:///D:\Diplome\Jo%C5%BEi%20Omahen-probioti%C4%8Dni%20izdelki%20Lek\Maldi%20rezultati\89%20izolatov%2020190426%20Bruker%20Daltonik%20MALDI%20Biotyper%20Classification%20Results.htm#ID0EA) | *L. reuteri* | *n.d.* | |
|  | 10c | *Lactobacillus reuteri* | [2.144](file:///D:\Diplome\Jo%C5%BEi%20Omahen-probioti%C4%8Dni%20izdelki%20Lek\Maldi%20rezultati\89%20izolatov%2020190426%20Bruker%20Daltonik%20MALDI%20Biotyper%20Classification%20Results.htm#ID0EA) | *L. reuteri* | *n.d.* | |
| 11 | 11a | *Bifidobacterium breve* | [2.155](file:///D:\Diplome\Jo%C5%BEi%20Omahen-probioti%C4%8Dni%20izdelki%20Lek\Maldi%20rezultati\89%20izolatov%2020190426%20Bruker%20Daltonik%20MALDI%20Biotyper%20Classification%20Results.htm#ID0EA) | *B. breve* | *n.d.* | |
|  | 11b | *Bifidobacterium animalis* | [2.109](file:///D:\Diplome\Jo%C5%BEi%20Omahen-probioti%C4%8Dni%20izdelki%20Lek\Maldi%20rezultati\89%20izolatov%2020190426%20Bruker%20Daltonik%20MALDI%20Biotyper%20Classification%20Results.htm#ID0EA) | *B. lactis* | *n.d.* | |
|  | 11c | *Bifidobacterium breve* | [1.97](file:///D:\Diplome\Jo%C5%BEi%20Omahen-probioti%C4%8Dni%20izdelki%20Lek\Maldi%20rezultati\89%20izolatov%2020190426%20Bruker%20Daltonik%20MALDI%20Biotyper%20Classification%20Results.htm#ID0EA) | *B. breve* | *n.d.* | |
|  | 11d | *Bifidobacterium breve* | [2.213](file:///D:\Diplome\Jo%C5%BEi%20Omahen-probioti%C4%8Dni%20izdelki%20Lek\Maldi%20rezultati\89%20izolatov%2020190426%20Bruker%20Daltonik%20MALDI%20Biotyper%20Classification%20Results.htm#ID0EA) | *B. breve* | *n.d.* | |
|  | 11e | *Bifidobacterium breve* | [2.218](file:///D:\Diplome\Jo%C5%BEi%20Omahen-probioti%C4%8Dni%20izdelki%20Lek\Maldi%20rezultati\89%20izolatov%2020190426%20Bruker%20Daltonik%20MALDI%20Biotyper%20Classification%20Results.htm#ID0EA) | *B. breve* | *n.d.* | |
|  | 11f | *Bifidobacterium breve* | [2.189](file:///D:\Diplome\Jo%C5%BEi%20Omahen-probioti%C4%8Dni%20izdelki%20Lek\Maldi%20rezultati\89%20izolatov%2020190426%20Bruker%20Daltonik%20MALDI%20Biotyper%20Classification%20Results.htm#ID0EA) | *B. breve* | *n.d.* | |
|  | 11g | *Bifidobacterium breve* | [2.261](file:///D:\Diplome\Jo%C5%BEi%20Omahen-probioti%C4%8Dni%20izdelki%20Lek\Maldi%20rezultati\89%20izolatov%2020190426%20Bruker%20Daltonik%20MALDI%20Biotyper%20Classification%20Results.htm#ID0EA) | *B. breve* | *n.d.* | |
|  | 11h | *Bifidobacterium breve* | [2.246](file:///D:\Diplome\Jo%C5%BEi%20Omahen-probioti%C4%8Dni%20izdelki%20Lek\Maldi%20rezultati\89%20izolatov%2020190426%20Bruker%20Daltonik%20MALDI%20Biotyper%20Classification%20Results.htm#ID0EA) | *B. breve* | *n.d.* | |
|  | 11i | *Bifidobacterium breve* | [1.744](file:///D:\Diplome\Jo%C5%BEi%20Omahen-probioti%C4%8Dni%20izdelki%20Lek\Maldi%20rezultati\89%20izolatov%2020190426%20Bruker%20Daltonik%20MALDI%20Biotyper%20Classification%20Results.htm#ID0EA) | *B. breve* | *n.d.* | |
|  | 11j | *Bifidobacterium breve* | [1.748](file:///D:\Diplome\Jo%C5%BEi%20Omahen-probioti%C4%8Dni%20izdelki%20Lek\Maldi%20rezultati\89%20izolatov%2020190426%20Bruker%20Daltonik%20MALDI%20Biotyper%20Classification%20Results.htm#ID0EA) | *B. breve* | *n.d.* | |
|  | 11k | *Bifidobacterium breve* | [2.265](file:///D:\Diplome\Jo%C5%BEi%20Omahen-probioti%C4%8Dni%20izdelki%20Lek\Maldi%20rezultati\89%20izolatov%2020190426%20Bruker%20Daltonik%20MALDI%20Biotyper%20Classification%20Results.htm#ID0EA) | *B. breve* | *n.d.* | |
|  | 11l | *Bifidobacterium breve* | [2.284](file:///D:\Diplome\Jo%C5%BEi%20Omahen-probioti%C4%8Dni%20izdelki%20Lek\Maldi%20rezultati\89%20izolatov%2020190426%20Bruker%20Daltonik%20MALDI%20Biotyper%20Classification%20Results.htm#ID0EA) | *B. breve* | *n.d.* | |
|  | 11m | [*Lactobacillus acidophilus*](file:///D:\Diplome\Jo%C5%BEi%20Omahen-probioti%C4%8Dni%20izdelki%20Lek\Maldi%20rezultati\89%20izolatov%2020190426%20Bruker%20Daltonik%20MALDI%20Biotyper%20Classification%20Results.htm#ID0EWCA) | [2.364](file:///D:\Diplome\Jo%C5%BEi%20Omahen-probioti%C4%8Dni%20izdelki%20Lek\Maldi%20rezultati\89%20izolatov%2020190426%20Bruker%20Daltonik%20MALDI%20Biotyper%20Classification%20Results.htm#ID0EA) | *L. acidophilus* | *n.d.* | |
|  | 11n | *Saccharomyces cerevisiae* | [1.894](file:///D:\Diplome\Jo%C5%BEi%20Omahen-probioti%C4%8Dni%20izdelki%20Lek\Maldi%20rezultati\89%20izolatov%2020190426%20Bruker%20Daltonik%20MALDI%20Biotyper%20Classification%20Results.htm#ID0EA) | *S. cerevisiae* | *n.d.* | |
|  | 11o | [*Lactobacillus acidophilus*](file:///D:\Diplome\Jo%C5%BEi%20Omahen-probioti%C4%8Dni%20izdelki%20Lek\Maldi%20rezultati\89%20izolatov%2020190426%20Bruker%20Daltonik%20MALDI%20Biotyper%20Classification%20Results.htm#ID0EWCA) | [2.344](file:///D:\Diplome\Jo%C5%BEi%20Omahen-probioti%C4%8Dni%20izdelki%20Lek\Maldi%20rezultati\89%20izolatov%2020190426%20Bruker%20Daltonik%20MALDI%20Biotyper%20Classification%20Results.htm#ID0EA) | *L. acidophilus* | *n.d.* | |
|  | 11p | *Saccharomyces cerevisiae* | [1.802](file:///D:\Diplome\Jo%C5%BEi%20Omahen-probioti%C4%8Dni%20izdelki%20Lek\Maldi%20rezultati\89%20izolatov%2020190426%20Bruker%20Daltonik%20MALDI%20Biotyper%20Classification%20Results.htm#ID0EA) | *S. cerevisiae* | *n.d.* | |
|  | 11r | [*Lactobacillus acidophilus*](file:///D:\Diplome\Jo%C5%BEi%20Omahen-probioti%C4%8Dni%20izdelki%20Lek\Maldi%20rezultati\89%20izolatov%2020190426%20Bruker%20Daltonik%20MALDI%20Biotyper%20Classification%20Results.htm#ID0E0HB0CA) | [2.318](file:///D:\Diplome\Jo%C5%BEi%20Omahen-probioti%C4%8Dni%20izdelki%20Lek\Maldi%20rezultati\89%20izolatov%2020190426%20Bruker%20Daltonik%20MALDI%20Biotyper%20Classification%20Results.htm#ID0EA) | *L. acidophilus* | *n.d.* | |
|  | 11s | *Saccharomyces cerevisiae* | [1.77](file:///D:\Diplome\Jo%C5%BEi%20Omahen-probioti%C4%8Dni%20izdelki%20Lek\Maldi%20rezultati\89%20izolatov%2020190426%20Bruker%20Daltonik%20MALDI%20Biotyper%20Classification%20Results.htm#ID0EA) | *S. cerevisiae* | *n.d.* | |
|  | 11t | *Saccharomyces cerevisiae* | [1.948](file:///D:\Diplome\Jo%C5%BEi%20Omahen-probioti%C4%8Dni%20izdelki%20Lek\Maldi%20rezultati\89%20izolatov%2020190426%20Bruker%20Daltonik%20MALDI%20Biotyper%20Classification%20Results.htm#ID0EA) | *S. cerevisiae* | *n.d.* | |
|  | 11u | *Saccharomyces cerevisiae* | [2.124](file:///D:\Diplome\Jo%C5%BEi%20Omahen-probioti%C4%8Dni%20izdelki%20Lek\Maldi%20rezultati\89%20izolatov%2020190426%20Bruker%20Daltonik%20MALDI%20Biotyper%20Classification%20Results.htm#ID0EA) | *S. cerevisiae* | *n.d.* | |
|  | 11v | *Saccharomyces cerevisiae* | [1.855](file:///D:\Diplome\Jo%C5%BEi%20Omahen-probioti%C4%8Dni%20izdelki%20Lek\Maldi%20rezultati\89%20izolatov%2020190426%20Bruker%20Daltonik%20MALDI%20Biotyper%20Classification%20Results.htm#ID0EA) | *S. cerevisiae* | *n.d.* | |
| 12 | 12a | *Enterococcus faecium* | [2.033](file:///D:\Diplome\Jo%C5%BEi%20Omahen-probioti%C4%8Dni%20izdelki%20Lek\Maldi%20rezultati\Petra%20LEK%20Bruker%20Daltonik%20MALDI%20Biotyper%20Classification%20Results.htm#ID0EA) | *E. faecium* | *n.d.* | |
|  | 12b | *Enterococcus faecium* | [1.779](file:///D:\Diplome\Jo%C5%BEi%20Omahen-probioti%C4%8Dni%20izdelki%20Lek\Maldi%20rezultati\Petra%20LEK%20Bruker%20Daltonik%20MALDI%20Biotyper%20Classification%20Results.htm#ID0EA) | *E. faecium* | *n.d.* | |
|  | 12c | *Enterococcus faecium* | [1.974](file:///D:\Diplome\Jo%C5%BEi%20Omahen-probioti%C4%8Dni%20izdelki%20Lek\Maldi%20rezultati\Petra%20LEK%20Bruker%20Daltonik%20MALDI%20Biotyper%20Classification%20Results.htm#ID0EA) | *E. faecium* | *n.d.* | |
|  | 12d | *Bifidobacterium animalis* | [1.925](file:///D:\Diplome\Jo%C5%BEi%20Omahen-probioti%C4%8Dni%20izdelki%20Lek\Maldi%20rezultati\Petra%20LEK%20Bruker%20Daltonik%20MALDI%20Biotyper%20Classification%20Results.htm#ID0EA) | *B. lactis* | *n.d.* | |
|  | 12e | *Bifidobacterium animalis* | [1.716](file:///D:\Diplome\Jo%C5%BEi%20Omahen-probioti%C4%8Dni%20izdelki%20Lek\Maldi%20rezultati\Petra%20LEK%20Bruker%20Daltonik%20MALDI%20Biotyper%20Classification%20Results.htm#ID0EA) | *B. lactis* | *n.d.* | |
|  | 12f | *Bifidobacterium animalis* | [2.146](file:///D:\Diplome\Jo%C5%BEi%20Omahen-probioti%C4%8Dni%20izdelki%20Lek\Maldi%20rezultati\Petra%20LEK%20Bruker%20Daltonik%20MALDI%20Biotyper%20Classification%20Results.htm#ID0EA) | *B. lactis* | *n.d.* | |
|  | 12g | [*Lactobacillus acidophilus*](file:///D:\Diplome\Jo%C5%BEi%20Omahen-probioti%C4%8Dni%20izdelki%20Lek\Maldi%20rezultati\Petra%20LEK%20Bruker%20Daltonik%20MALDI%20Biotyper%20Classification%20Results.htm#ID0EUCA) | [2.321](file:///D:\Diplome\Jo%C5%BEi%20Omahen-probioti%C4%8Dni%20izdelki%20Lek\Maldi%20rezultati\Petra%20LEK%20Bruker%20Daltonik%20MALDI%20Biotyper%20Classification%20Results.htm#ID0EA) | *L. acidophilus* | *n.d.* | |
|  | 12h | *Lactobacillus paracasei* | [2.123](file:///D:\Diplome\Jo%C5%BEi%20Omahen-probioti%C4%8Dni%20izdelki%20Lek\Maldi%20rezultati\Petra%20LEK%20Bruker%20Daltonik%20MALDI%20Biotyper%20Classification%20Results.htm#ID0EA) | *L. paracasei* | *n.d.* | |
|  | 12i | *Lactobacillus paracasei* | [2.28](file:///D:\Diplome\Jo%C5%BEi%20Omahen-probioti%C4%8Dni%20izdelki%20Lek\Maldi%20rezultati\Petra%20LEK%20Bruker%20Daltonik%20MALDI%20Biotyper%20Classification%20Results.htm#ID0EA) | *L. paracasei* | *n.d.* | |
|  | 12j | *Lactobacillus salivarius* | [2.186](file:///D:\Diplome\Jo%C5%BEi%20Omahen-probioti%C4%8Dni%20izdelki%20Lek\Maldi%20rezultati\Petra%20LEK%20Bruker%20Daltonik%20MALDI%20Biotyper%20Classification%20Results.htm#ID0EA) | *L. salivarius* | *n.d.* | |
|  | 12k | [*Lactobacillus acidophilus*](file:///D:\Diplome\Jo%C5%BEi%20Omahen-probioti%C4%8Dni%20izdelki%20Lek\Maldi%20rezultati\Petra%20LEK%20Bruker%20Daltonik%20MALDI%20Biotyper%20Classification%20Results.htm#ID0EUCA) | [1.913](file:///D:\Diplome\Jo%C5%BEi%20Omahen-probioti%C4%8Dni%20izdelki%20Lek\Maldi%20rezultati\Petra%20LEK%20Bruker%20Daltonik%20MALDI%20Biotyper%20Classification%20Results.htm#ID0EA) | *L. acidophilus* | *n.d.* | |
|  | 12l | *Lactobacillus salivarius* | [2.058](file:///D:\Diplome\Jo%C5%BEi%20Omahen-probioti%C4%8Dni%20izdelki%20Lek\Maldi%20rezultati\Petra%20LEK%20Bruker%20Daltonik%20MALDI%20Biotyper%20Classification%20Results.htm#ID0EA) | *L. salivarius* | *n.d.* | |
|  | 12m | [*Lactobacillus acidophilus*](file:///D:\Diplome\Jo%C5%BEi%20Omahen-probioti%C4%8Dni%20izdelki%20Lek\Maldi%20rezultati\Petra%20LEK%20Bruker%20Daltonik%20MALDI%20Biotyper%20Classification%20Results.htm#ID0EUCA) | [2.265](file:///D:\Diplome\Jo%C5%BEi%20Omahen-probioti%C4%8Dni%20izdelki%20Lek\Maldi%20rezultati\Petra%20LEK%20Bruker%20Daltonik%20MALDI%20Biotyper%20Classification%20Results.htm#ID0EA) | *L. acidophilus* | *n.d.* | |
|  | 12n | *Lactobacillus salivarius* | [2.219](file:///D:\Diplome\Jo%C5%BEi%20Omahen-probioti%C4%8Dni%20izdelki%20Lek\Maldi%20rezultati\Petra%20LEK%20Bruker%20Daltonik%20MALDI%20Biotyper%20Classification%20Results.htm#ID0EA) | *L. salivarius* | *n.d.* | |
|  | 12o | *Lactobacillus paracasei* | [2.097](file:///D:\Diplome\Jo%C5%BEi%20Omahen-probioti%C4%8Dni%20izdelki%20Lek\Maldi%20rezultati\Petra%20LEK%20Bruker%20Daltonik%20MALDI%20Biotyper%20Classification%20Results.htm#ID0EA) | *L. paracasei* | *n.d.* | |
|  | 12p | *Lactococcus lactis* | [2.251](file:///D:\Diplome\Jo%C5%BEi%20Omahen-probioti%C4%8Dni%20izdelki%20Lek\Maldi%20rezultati\Petra%20LEK%20Bruker%20Daltonik%20MALDI%20Biotyper%20Classification%20Results.htm#ID0EA) | *Lc. lactis* | *n.d.* | |
|  | 12r | *Enterococcus faecium* | [1.855](file:///D:\Diplome\Jo%C5%BEi%20Omahen-probioti%C4%8Dni%20izdelki%20Lek\Maldi%20rezultati\Petra%20LEK%20Bruker%20Daltonik%20MALDI%20Biotyper%20Classification%20Results.htm#ID0EA) | *E. faecium* | *n.d.* | |
|  | 12s | *Enterococcus faecium* | [1.94](file:///D:\Diplome\Jo%C5%BEi%20Omahen-probioti%C4%8Dni%20izdelki%20Lek\Maldi%20rezultati\Petra%20LEK%20Bruker%20Daltonik%20MALDI%20Biotyper%20Classification%20Results.htm#ID0EA) | *E. faecium* | *n.d.* | |
| 13 | 13a | ***no peaks found*** | [< 0](file:///D:\Diplome\Jo%C5%BEi%20Omahen-probioti%C4%8Dni%20izdelki%20Lek\Maldi%20rezultati\Petra%20LEK%20Bruker%20Daltonik%20MALDI%20Biotyper%20Classification%20Results.htm#ID0EA) | *E. faecium* | *n.d.* | |
|  | 13b | ***no peaks found*** | [< 0](file:///D:\Diplome\Jo%C5%BEi%20Omahen-probioti%C4%8Dni%20izdelki%20Lek\Maldi%20rezultati\Petra%20LEK%20Bruker%20Daltonik%20MALDI%20Biotyper%20Classification%20Results.htm#ID0EA) | *E. faecium* | *n.d.* | |
|  | 13c | *Enterococcus faecium* | [1.894](file:///D:\Diplome\Jo%C5%BEi%20Omahen-probioti%C4%8Dni%20izdelki%20Lek\Maldi%20rezultati\Petra%20LEK%20Bruker%20Daltonik%20MALDI%20Biotyper%20Classification%20Results.htm#ID0EA) | *E. faecium* | *n.d.* | |
|  | 13d | *Lactobacillus plantarum* | [1.731](file:///D:\Diplome\Jo%C5%BEi%20Omahen-probioti%C4%8Dni%20izdelki%20Lek\Maldi%20rezultati\Petra%20LEK%20Bruker%20Daltonik%20MALDI%20Biotyper%20Classification%20Results.htm#ID0EA) | *L. plantarum* | *n.d.* | |
|  | 13e | *Lactobacillus paracasei* | [1.796](file:///D:\Diplome\Jo%C5%BEi%20Omahen-probioti%C4%8Dni%20izdelki%20Lek\Maldi%20rezultati\Petra%20LEK%20Bruker%20Daltonik%20MALDI%20Biotyper%20Classification%20Results.htm#ID0EA) | *L. paracasei* | *n.d.* | |
|  | 13f | [*Lactobacillus acidophilus*](file:///D:\Diplome\Jo%C5%BEi%20Omahen-probioti%C4%8Dni%20izdelki%20Lek\Maldi%20rezultati\Petra%20LEK%20Bruker%20Daltonik%20MALDI%20Biotyper%20Classification%20Results.htm#ID0EUCA) | [2.32](file:///D:\Diplome\Jo%C5%BEi%20Omahen-probioti%C4%8Dni%20izdelki%20Lek\Maldi%20rezultati\Petra%20LEK%20Bruker%20Daltonik%20MALDI%20Biotyper%20Classification%20Results.htm#ID0EA) | *L. acidophilus* | *n.d.* | |
|  | 13g | [*Lactobacillus acidophilus*](file:///D:\Diplome\Jo%C5%BEi%20Omahen-probioti%C4%8Dni%20izdelki%20Lek\Maldi%20rezultati\Petra%20LEK%20Bruker%20Daltonik%20MALDI%20Biotyper%20Classification%20Results.htm#ID0EUCA) | [1.919](file:///D:\Diplome\Jo%C5%BEi%20Omahen-probioti%C4%8Dni%20izdelki%20Lek\Maldi%20rezultati\Petra%20LEK%20Bruker%20Daltonik%20MALDI%20Biotyper%20Classification%20Results.htm#ID0EA) | *L. acidophilus* | *n.d.* | |
|  | 13h | *Lactobacillus zeae* | [1.956](file:///D:\Diplome\Jo%C5%BEi%20Omahen-probioti%C4%8Dni%20izdelki%20Lek\Maldi%20rezultati\Petra%20LEK%20Bruker%20Daltonik%20MALDI%20Biotyper%20Classification%20Results.htm#ID0EA) | *L. rhamnosus* | *L. rhamnosus* | |
|  | 13i | *Lactobacillus plantarum* | [2.274](file:///D:\Diplome\Jo%C5%BEi%20Omahen-probioti%C4%8Dni%20izdelki%20Lek\Maldi%20rezultati\Petra%20LEK%20Bruker%20Daltonik%20MALDI%20Biotyper%20Classification%20Results.htm#ID0EA) | *L. plantarum* | *n.d.* | |
|  | 13j | *Lactobacillus plantarum* | [2.138](file:///D:\Diplome\Jo%C5%BEi%20Omahen-probioti%C4%8Dni%20izdelki%20Lek\Maldi%20rezultati\Petra%20LEK%20Bruker%20Daltonik%20MALDI%20Biotyper%20Classification%20Results.htm#ID0EA) | *L. plantarum* | *n.d.* | |
|  | 13k | *Lactobacillus plantarum* | [2.207](file:///D:\Diplome\Jo%C5%BEi%20Omahen-probioti%C4%8Dni%20izdelki%20Lek\Maldi%20rezultati\Petra%20LEK%20Bruker%20Daltonik%20MALDI%20Biotyper%20Classification%20Results.htm#ID0EA) | *L. plantarum* | *n.d.* | |
|  | 13l | *Lactobacillus plantarum* | [1.989](file:///D:\Diplome\Jo%C5%BEi%20Omahen-probioti%C4%8Dni%20izdelki%20Lek\Maldi%20rezultati\Petra%20LEK%20Bruker%20Daltonik%20MALDI%20Biotyper%20Classification%20Results.htm#ID0EA) | *L. plantarum* | *n.d.* | |
|  | 13m | *Lactobacillus salivarius* | [2.085](file:///D:\Diplome\Jo%C5%BEi%20Omahen-probioti%C4%8Dni%20izdelki%20Lek\Maldi%20rezultati\Petra%20LEK%20Bruker%20Daltonik%20MALDI%20Biotyper%20Classification%20Results.htm#ID0EA) | *L. salivarius* | *n.d.* | |
|  | 13n | [*Lactobacillus acidophilus*](file:///D:\Diplome\Jo%C5%BEi%20Omahen-probioti%C4%8Dni%20izdelki%20Lek\Maldi%20rezultati\Petra%20LEK%20Bruker%20Daltonik%20MALDI%20Biotyper%20Classification%20Results.htm#ID0E0BB0CA) | [2.388](file:///D:\Diplome\Jo%C5%BEi%20Omahen-probioti%C4%8Dni%20izdelki%20Lek\Maldi%20rezultati\Petra%20LEK%20Bruker%20Daltonik%20MALDI%20Biotyper%20Classification%20Results.htm#ID0EA) | *L. acidophilus* | *n.d.* | |
|  | 13o | [*Lactobacillus acidophilus*](file:///D:\Diplome\Jo%C5%BEi%20Omahen-probioti%C4%8Dni%20izdelki%20Lek\Maldi%20rezultati\Petra%20LEK%20Bruker%20Daltonik%20MALDI%20Biotyper%20Classification%20Results.htm#ID0EUCA) | [2.395](file:///D:\Diplome\Jo%C5%BEi%20Omahen-probioti%C4%8Dni%20izdelki%20Lek\Maldi%20rezultati\Petra%20LEK%20Bruker%20Daltonik%20MALDI%20Biotyper%20Classification%20Results.htm#ID0EA) | *L. acidophilus* | *n.d.* | |
|  | 13p | [*Lactobacillus acidophilus*](file:///D:\Diplome\Jo%C5%BEi%20Omahen-probioti%C4%8Dni%20izdelki%20Lek\Maldi%20rezultati\Petra%20LEK%20Bruker%20Daltonik%20MALDI%20Biotyper%20Classification%20Results.htm#ID0EUCA) | [2.267](file:///D:\Diplome\Jo%C5%BEi%20Omahen-probioti%C4%8Dni%20izdelki%20Lek\Maldi%20rezultati\Petra%20LEK%20Bruker%20Daltonik%20MALDI%20Biotyper%20Classification%20Results.htm#ID0EA) | *L. acidophilus* | *n.d.* | |
|  | 13r | [*Lactobacillus acidophilus*](file:///D:\Diplome\Jo%C5%BEi%20Omahen-probioti%C4%8Dni%20izdelki%20Lek\Maldi%20rezultati\Petra%20LEK%20Bruker%20Daltonik%20MALDI%20Biotyper%20Classification%20Results.htm#ID0EUCA) | [2.197](file:///D:\Diplome\Jo%C5%BEi%20Omahen-probioti%C4%8Dni%20izdelki%20Lek\Maldi%20rezultati\Petra%20LEK%20Bruker%20Daltonik%20MALDI%20Biotyper%20Classification%20Results.htm#ID0EA) | *L. acidophilus* | *n.d.* | |
|  | 13s | ***not reliable identification*** | [1.556](file:///D:\Diplome\Jo%C5%BEi%20Omahen-probioti%C4%8Dni%20izdelki%20Lek\Maldi%20rezultati\Petra%20LEK%20Bruker%20Daltonik%20MALDI%20Biotyper%20Classification%20Results.htm#ID0EA) | *L. plantarum* | *n.d.* | |
|  | 13t | *Lactobacillus plantarum* | [1.735](file:///D:\Diplome\Jo%C5%BEi%20Omahen-probioti%C4%8Dni%20izdelki%20Lek\Maldi%20rezultati\Petra%20LEK%20Bruker%20Daltonik%20MALDI%20Biotyper%20Classification%20Results.htm#ID0EA) | *L. plantarum* | *n.d.* | |
|  | 13u | [*Lactobacillus acidophilus*](file:///D:\Diplome\Jo%C5%BEi%20Omahen-probioti%C4%8Dni%20izdelki%20Lek\Maldi%20rezultati\Petra%20LEK%20Bruker%20Daltonik%20MALDI%20Biotyper%20Classification%20Results.htm#ID0EUCA) | [2.306](file:///D:\Diplome\Jo%C5%BEi%20Omahen-probioti%C4%8Dni%20izdelki%20Lek\Maldi%20rezultati\Petra%20LEK%20Bruker%20Daltonik%20MALDI%20Biotyper%20Classification%20Results.htm#ID0EA) | *L. acidophilus* | *n.d.* | |
|  | 13v | [*Lactobacillus acidophilus*](file:///D:\Diplome\Jo%C5%BEi%20Omahen-probioti%C4%8Dni%20izdelki%20Lek\Maldi%20rezultati\Petra%20LEK%20Bruker%20Daltonik%20MALDI%20Biotyper%20Classification%20Results.htm#ID0EUCA) | [2.433](file:///D:\Diplome\Jo%C5%BEi%20Omahen-probioti%C4%8Dni%20izdelki%20Lek\Maldi%20rezultati\Petra%20LEK%20Bruker%20Daltonik%20MALDI%20Biotyper%20Classification%20Results.htm#ID0EA) | *L. acidophilus* | *n.d.* | |
|  | 13a2 | *Bifidobacterium animalis* | [2.05](file:///D:\Diplome\Jo%C5%BEi%20Omahen-probioti%C4%8Dni%20izdelki%20Lek\Maldi%20rezultati\Petra%20LEK%20Bruker%20Daltonik%20MALDI%20Biotyper%20Classification%20Results.htm#ID0EA) | *B. lactis* | *n.d.* | |
|  | 13b2 | *Bifidobacterium animalis* | [1.92](file:///D:\Diplome\Jo%C5%BEi%20Omahen-probioti%C4%8Dni%20izdelki%20Lek\Maldi%20rezultati\Petra%20LEK%20Bruker%20Daltonik%20MALDI%20Biotyper%20Classification%20Results.htm#ID0EA) | *B. lactis* | *n.d.* | |
|  | 13c2 | *Bifidobacterium animalis* | [1.988](file:///D:\Diplome\Jo%C5%BEi%20Omahen-probioti%C4%8Dni%20izdelki%20Lek\Maldi%20rezultati\Petra%20LEK%20Bruker%20Daltonik%20MALDI%20Biotyper%20Classification%20Results.htm#ID0EA) | *B. lactis* | *n.d.* | |
|  | 13d2 | *Bifidobacterium animalis* | [2.147](file:///D:\Diplome\Jo%C5%BEi%20Omahen-probioti%C4%8Dni%20izdelki%20Lek\Maldi%20rezultati\Petra%20LEK%20Bruker%20Daltonik%20MALDI%20Biotyper%20Classification%20Results.htm#ID0EA) | *B. lactis* | *n.d.* | |
|  | 13e2 | *Bifidobacterium animalis* | [1.94](file:///D:\Diplome\Jo%C5%BEi%20Omahen-probioti%C4%8Dni%20izdelki%20Lek\Maldi%20rezultati\Petra%20LEK%20Bruker%20Daltonik%20MALDI%20Biotyper%20Classification%20Results.htm#ID0EA) | *B. lactis* | *n.d.* | |
|  | 13f2 | *Bifidobacterium animalis* | [1.718](file:///D:\Diplome\Jo%C5%BEi%20Omahen-probioti%C4%8Dni%20izdelki%20Lek\Maldi%20rezultati\Petra%20LEK%20Bruker%20Daltonik%20MALDI%20Biotyper%20Classification%20Results.htm#ID0EA) | *B. lactis* | *n.d.* | |
|  | 13g2 | *Bifidobacterium animalis* | [1.74](file:///D:\Diplome\Jo%C5%BEi%20Omahen-probioti%C4%8Dni%20izdelki%20Lek\Maldi%20rezultati\Petra%20LEK%20Bruker%20Daltonik%20MALDI%20Biotyper%20Classification%20Results.htm#ID0EA) | *B. lactis* | *n.d.* | |
|  | 13h2 | *Bifidobacterium animalis* | [2.025](file:///D:\Diplome\Jo%C5%BEi%20Omahen-probioti%C4%8Dni%20izdelki%20Lek\Maldi%20rezultati\Petra%20LEK%20Bruker%20Daltonik%20MALDI%20Biotyper%20Classification%20Results.htm#ID0EA) | *B. lactis* | *n.d.* | |
|  | 13i2 | *Bifidobacterium animalis* | [2.202](file:///D:\Diplome\Jo%C5%BEi%20Omahen-probioti%C4%8Dni%20izdelki%20Lek\Maldi%20rezultati\Petra%20LEK%20Bruker%20Daltonik%20MALDI%20Biotyper%20Classification%20Results.htm#ID0EA) | *B. lactis* | *n.d.* | |
| 14 | 14a | ***not reliable identification*** | [1.68](file:///D:\Diplome\Jo%C5%BEi%20Omahen-probioti%C4%8Dni%20izdelki%20Lek\Maldi%20rezultati\Bruker%20Daltonik%20MALDI%2018.6..htm#ID0EA) | *L. rhamnosus* | *n.d.* | |
|  | 14b | *Lactobacillus rhamnosus* | [1.818](file:///D:\Diplome\Jo%C5%BEi%20Omahen-probioti%C4%8Dni%20izdelki%20Lek\Maldi%20rezultati\Bruker%20Daltonik%20MALDI%2018.6..htm#ID0EA) | *L. rhamnosus* | *n.d.* | |
|  | 14c | *Lactobacillus rhamnosus* | [1.821](file:///D:\Diplome\Jo%C5%BEi%20Omahen-probioti%C4%8Dni%20izdelki%20Lek\Maldi%20rezultati\Bruker%20Daltonik%20MALDI%2018.6..htm#ID0EA) | *L. rhamnosus* | *n.d.* | |
| 15 | 15a | *Enterococcus faecium* | [2.048](file:///D:\Diplome\Jo%C5%BEi%20Omahen-probioti%C4%8Dni%20izdelki%20Lek\Maldi%20rezultati\Bruker%20Daltonik%20MALDI%2018.6..htm#ID0EA) | *E. faecium* | *n.d.* | |
|  | 15b | *Enterococcus faecium* | [2.129](file:///D:\Diplome\Jo%C5%BEi%20Omahen-probioti%C4%8Dni%20izdelki%20Lek\Maldi%20rezultati\Bruker%20Daltonik%20MALDI%2018.6..htm#ID0EA) | *E. faecium* | *n.d.* | |
|  | 15c | *Enterococcus faecium* | [2.08](file:///D:\Diplome\Jo%C5%BEi%20Omahen-probioti%C4%8Dni%20izdelki%20Lek\Maldi%20rezultati\Bruker%20Daltonik%20MALDI%2018.6..htm#ID0EA) | *E. faecium* | *n.d.* | |
|  | 15d | *Lactobacillus gasseri* | [2.368](file:///D:\Diplome\Jo%C5%BEi%20Omahen-probioti%C4%8Dni%20izdelki%20Lek\Maldi%20rezultati\Bruker%20Daltonik%20MALDI%2018.6..htm#ID0EA) | *L. gasseri* | *n.d.* | |
|  | 15e | *Lactobacillus gasseri* | [2.314](file:///D:\Diplome\Jo%C5%BEi%20Omahen-probioti%C4%8Dni%20izdelki%20Lek\Maldi%20rezultati\Bruker%20Daltonik%20MALDI%2018.6..htm#ID0EA) | *L. gasseri* | *n.d.* | |
|  | 15f | *Lactobacillus gasseri* | [2.349](file:///D:\Diplome\Jo%C5%BEi%20Omahen-probioti%C4%8Dni%20izdelki%20Lek\Maldi%20rezultati\Bruker%20Daltonik%20MALDI%2018.6..htm#ID0EA) | *L. gasseri* | *n.d.* | |
|  | 15g | *Bifidobacterium longum infantis* | [2.056](file:///D:\Diplome\Jo%C5%BEi%20Omahen-probioti%C4%8Dni%20izdelki%20Lek\Maldi%20rezultati\Bruker%20Daltonik%20MALDI%2018.6..htm#ID0EA) | *B. infantis* | *n.d.* | |
|  | 15h | *Bifidobacterium longum infantis* | [2.155](file:///D:\Diplome\Jo%C5%BEi%20Omahen-probioti%C4%8Dni%20izdelki%20Lek\Maldi%20rezultati\Bruker%20Daltonik%20MALDI%2018.6..htm#ID0EA) | *B. infantis* | *n.d.* | |
|  | 15i | *Bifidobacterium longum ali infantis* | [2.17](file:///D:\Diplome\Jo%C5%BEi%20Omahen-probioti%C4%8Dni%20izdelki%20Lek\Maldi%20rezultati\Bruker%20Daltonik%20MALDI%2018.6..htm#ID0EA) | *B. infantis* | *n.d.* | |
| 16 | 16a | ***no peaks found*** | [< 0](file:///D:\Diplome\Jo%C5%BEi%20Omahen-probioti%C4%8Dni%20izdelki%20Lek\Maldi%20rezultati\Petra%20LEK%20Bruker%20Daltonik%20MALDI%20Biotyper%20Classification%20Results.htm#ID0EA) | *B. lactis* | *n.d.* | |
|  | 16b | *Bifidobacterium animalis* | [2.314](file:///D:\Diplome\Jo%C5%BEi%20Omahen-probioti%C4%8Dni%20izdelki%20Lek\Maldi%20rezultati\Petra%20LEK%20Bruker%20Daltonik%20MALDI%20Biotyper%20Classification%20Results.htm#ID0EA) | *B. lactis* | *n.d.* | |
|  | 16c | *Bifidobacterium animalis* | [2.316](file:///D:\Diplome\Jo%C5%BEi%20Omahen-probioti%C4%8Dni%20izdelki%20Lek\Maldi%20rezultati\Petra%20LEK%20Bruker%20Daltonik%20MALDI%20Biotyper%20Classification%20Results.htm#ID0EA) | *B. lactis* | *n.d.* | |
|  | 16d | ***no peaks found*** | [< 0](file:///D:\Diplome\Jo%C5%BEi%20Omahen-probioti%C4%8Dni%20izdelki%20Lek\Maldi%20rezultati\Petra%20LEK%20Bruker%20Daltonik%20MALDI%20Biotyper%20Classification%20Results.htm#ID0EA) | *L. acidophilus* | *n.d.* | |
|  | 16e | ***no peaks found*** | [< 0](file:///D:\Diplome\Jo%C5%BEi%20Omahen-probioti%C4%8Dni%20izdelki%20Lek\Maldi%20rezultati\Petra%20LEK%20Bruker%20Daltonik%20MALDI%20Biotyper%20Classification%20Results.htm#ID0EA) | *L. acidophilus* | *n.d.* | |
|  | 16f | ***no peaks found*** | [< 0](file:///D:\Diplome\Jo%C5%BEi%20Omahen-probioti%C4%8Dni%20izdelki%20Lek\Maldi%20rezultati\Petra%20LEK%20Bruker%20Daltonik%20MALDI%20Biotyper%20Classification%20Results.htm#ID0EA) | *L. acidophilus* | *n.d.* | |
| 17 | 17a | *Lactobacillus plantarum* | [2.053](file:///D:\Diplome\Jo%C5%BEi%20Omahen-probioti%C4%8Dni%20izdelki%20Lek\Maldi%20rezultati\Bruker%20Daltonik%20MALDI%2018.6..htm#ID0EA) | *L. plantarum* | *n.d.* | |
|  | 17b | *Lactobacillus plantarum* | [1.886](file:///D:\Diplome\Jo%C5%BEi%20Omahen-probioti%C4%8Dni%20izdelki%20Lek\Maldi%20rezultati\Bruker%20Daltonik%20MALDI%2018.6..htm#ID0EA) | *L. plantarum* | *n.d.* | |
|  | 17c | ***not reliable identification*** | [1.664](file:///D:\Diplome\Jo%C5%BEi%20Omahen-probioti%C4%8Dni%20izdelki%20Lek\Maldi%20rezultati\Bruker%20Daltonik%20MALDI%2018.6..htm#ID0EA) | *n.d.* | *n.d.* | |
|  | 17d | [*Lactobacillus acidophilus*](file:///D:\Diplome\Jo%C5%BEi%20Omahen-probioti%C4%8Dni%20izdelki%20Lek\Maldi%20rezultati\Bruker%20Daltonik%20MALDI%2018.6..htm#ID0E5CA) | [2.188](file:///D:\Diplome\Jo%C5%BEi%20Omahen-probioti%C4%8Dni%20izdelki%20Lek\Maldi%20rezultati\Bruker%20Daltonik%20MALDI%2018.6..htm#ID0EA) | *L. acidophilus* | *n.d.* | |
|  | 17e | *Lactobacillus plantarum* | [1.778](file:///D:\Diplome\Jo%C5%BEi%20Omahen-probioti%C4%8Dni%20izdelki%20Lek\Maldi%20rezultati\Bruker%20Daltonik%20MALDI%2018.6..htm#ID0EA) | *L. plantarum* | *n.d.* | |
|  | 17f | *Lactobacillus plantarum* | [2.102](file:///D:\Diplome\Jo%C5%BEi%20Omahen-probioti%C4%8Dni%20izdelki%20Lek\Maldi%20rezultati\Bruker%20Daltonik%20MALDI%2018.6..htm#ID0EA) | *L. plantarum* | *n.d.* | |
|  | 17g | *Lactobacillus plantarum* | [2.009](file:///D:\Diplome\Jo%C5%BEi%20Omahen-probioti%C4%8Dni%20izdelki%20Lek\Maldi%20rezultati\Bruker%20Daltonik%20MALDI%2018.6..htm#ID0EA) | *L. plantarum* | *n.d.* | |
|  | 17h | *Lactobacillus plantarum* | [1.74](file:///D:\Diplome\Jo%C5%BEi%20Omahen-probioti%C4%8Dni%20izdelki%20Lek\Maldi%20rezultati\Bruker%20Daltonik%20MALDI%2018.6..htm#ID0EA) | *L. plantarum* | *n.d.* | |
|  | 17i | *Lactobacillus plantarum* | [1.913](file:///D:\Diplome\Jo%C5%BEi%20Omahen-probioti%C4%8Dni%20izdelki%20Lek\Maldi%20rezultati\Bruker%20Daltonik%20MALDI%2018.6..htm#ID0EA) | *L. plantarum* | *n.d.* | |
|  | 17j | *Lactobacillus plantarum* | [1.819](file:///D:\Diplome\Jo%C5%BEi%20Omahen-probioti%C4%8Dni%20izdelki%20Lek\Maldi%20rezultati\Bruker%20Daltonik%20MALDI%2018.6..htm#ID0EA) | *L. plantarum* | *n.d.* | |
|  | 17k | [*Lactobacillus acidophilus*](file:///D:\Diplome\Jo%C5%BEi%20Omahen-probioti%C4%8Dni%20izdelki%20Lek\Maldi%20rezultati\Bruker%20Daltonik%20MALDI%2018.6..htm#ID0E0FB0CA) | [2.224](file:///D:\Diplome\Jo%C5%BEi%20Omahen-probioti%C4%8Dni%20izdelki%20Lek\Maldi%20rezultati\Bruker%20Daltonik%20MALDI%2018.6..htm#ID0EA) | *L. acidophilus* | *n.d.* | |
|  | 17l | *Lactobacillus plantarum* | [2.025](file:///D:\Diplome\Jo%C5%BEi%20Omahen-probioti%C4%8Dni%20izdelki%20Lek\Maldi%20rezultati\Bruker%20Daltonik%20MALDI%2018.6..htm#ID0EA) | *L. plantarum* | *n.d.* | |
|  | 17m | *Lactobacillus plantarum* | [2.226](file:///D:\Diplome\Jo%C5%BEi%20Omahen-probioti%C4%8Dni%20izdelki%20Lek\Maldi%20rezultati\Bruker%20Daltonik%20MALDI%2018.6..htm#ID0EA) | *L. plantarum* | *n.d.* | |
|  | 17n | *Lactobacillus plantarum* | [1.958](file:///D:\Diplome\Jo%C5%BEi%20Omahen-probioti%C4%8Dni%20izdelki%20Lek\Maldi%20rezultati\Bruker%20Daltonik%20MALDI%2018.6..htm#ID0EA) | *L. plantarum* | *n.d.* | |

_________________________________________________________________­­­­

*Bac.. Bacillus; B.. Bifidobacterium; B. lactis, Bifidobacterium animalis* subsp. *lactis; B. infantis, B. longum* subsp. *infantis; E.. Enterococcus; L.. Lactobacillus* (*in accordance with new taxonomic classification.* Zheng *et al.* (2020). also *Lactocaseibacillus. Lactiplantibacillus. Limosilactobacillus. Ligilactobacillus*); *Lb. bulgaricus,* *Lb. delbrueckii* subsp*. bulgaricus; Lc.. Lactococcus; S. thermophilus, Streptococcus salivarius* subsp. *thermophilus;*

**Supplementary Table 4:** Overview of the MALDI-TOF MS (Biotyper) identification of 70 isolates (3 isolates per labelled strain) from 9 dietary supplements for children (products 18-26).

| **Result of identification by MALDI-TOF MS (Biotyper)** | **Score value ≥ 2.000** | **Score value 1.700-1.999** |
| --- | --- | --- |
| *B. animalis subsp. lactis* | 14 | 1 |
| *B. breve* | 2 | 2 |
| *B. longum* | 1 | 1 |
| *L. acidophilus* | 1 |  |
| *L. casei* |  | 1 |
| *L. gallinarum* |  | 2 |
| *L. paracasei* | 5 | 3 |
| *L. helveticus* |  | 1 |
| *L. reuteri* | 5 | 1 |
| *L. rhamnosus* | 13 | 6 |
| *L. zeae* |  | 7 |
| *S. thermophilus* |  | 1 |
| *Lc. lactis* | 3 |  |
